# Genomic surveillance identifies potential risk factors for SARS-CoV-2 transmission at a mid-sized university in a small rural town

**DOI:** 10.1038/s41598-023-34625-7

**Published:** 2023-05-16

**Authors:** Kimberly R. Andrews, Daniel D. New, Digpal S. Gour, Kane Francetich, Scott A. Minnich, Barrie D. Robison, Carolyn J. Hovde

**Affiliations:** 1 Institute for Interdisciplinary Data Sciences, University of Idaho, Moscow, ID 83844 USA; 2 Gritman Medical Center, Moscow, ID 83843 USA; 3 Department of Animal, Veterinary and Food Science, University of Idaho, Moscow, ID 83844 USA

**Keywords:** Health policy, Genetics research, Evolutionary genetics, Phylogenetics, Evolutionary biology, Genomics, Medical genetics

## Abstract

Understanding transmission dynamics of SARS-CoV-2 in institutions of higher education (IHEs) is important because these settings have potential for rapid viral spread. Here, we used genomic surveillance to retrospectively investigate transmission dynamics throughout the 2020–2021 academic year for the University of Idaho (“University”), a mid-sized IHE in a small rural town. We generated genome assemblies for 1168 SARS-CoV-2 samples collected during the academic year, representing 46.8% of positive samples collected from the University population and 49.8% of positive samples collected from the surrounding community (“Community”) at the local hospital during this time. Transmission dynamics differed for the University when compared to the Community, with more infection waves that lasted shorter lengths of time, potentially resulting from high-transmission congregate settings along with mitigation efforts implemented by the University to combat outbreaks. We found evidence for low transmission rates between the University and Community, with approximately 8% of transmissions into the Community originating from the University, and approximately 6% of transmissions into the University originating from the Community. Potential transmission risk factors identified for the University included congregate settings such as sorority and fraternity events and residences, holiday travel, and high caseloads in the surrounding community. Knowledge of these risk factors can help the University and other IHEs develop effective mitigation measures for SARS-CoV-2 and similar pathogens.

## Introduction

Transmission dynamics in institutions of higher education (IHEs) remain poorly understood for severe acute respiratory syndrome coronavirus 2 (SARS-CoV-2), the virus that causes coronavirus 2019 disease (COVID-19). IHEs have potential for rapid spread of SARS-CoV-2 because they have a young adult population predisposed to low disease severity, and a wide range of congregate settings such as on-campus housing, classrooms, and social gatherings. Furthermore, IHEs could drive transmission to surrounding communities, which can be more susceptible to severe disease than IHE populations^1–4^. Many IHEs reported outbreaks of COVID-19 shortly after the 2020–2021 academic year began^3,5–9^. Cases also increased rapidly during this time in US counties that had IHEs^1,3,10^, although increases were less severe in counties with IHEs that implemented mitigation measures including online instruction and SARS-CoV-2 surveillance testing^11^. Outbreaks in IHEs have been linked to a range of sources, including shared living accommodation, sorority and fraternity activities, and off-campus social events^5,8,12–14^. However, transmission dynamics can vary across IHEs, and the factors driving this variation are not well understood^3^. Furthermore, few studies have examined IHE transmission dynamics across an entire academic year, leaving questions about how risk factors could vary at different timepoints in the academic calendar.

One of the most powerful tools for understanding SARS-CoV-2 transmission dynamics is genomic surveillance^15–17^. This technique involves sequencing entire viral genomes from SARS-CoV-2-positive samples and using these genome sequences to track viral lineages over time. For example, genomic surveillance can help determine whether rising COVID-19 infections are driven by one or more super spreader events (e.g., large social gatherings or conferences), congregate living accommodations (e.g., dormitories or care homes), new introductions from outside sources (e.g., due to holiday travel), introduction of a new variant with increased infectivity (e.g., the Omicron variant), or other factors^17–22^. For IHEs, this information can be used to identify risk factors for increased transmission throughout the academic year, and to help develop effective mitigation measures for future outbreaks of COVID-19 or other pathogens with similar transmission mechanisms.

Here we used genomic surveillance to retrospectively characterize SARS-CoV-2 transmission dynamics throughout the 2020–2021 academic year at the University of Idaho (hereafter referred to as “University”), a mid-sized university in a small rural town. The University held in-person classes for the 2020–2021 academic year and implemented a variety of COVID-19 mitigation measures, including mandatory surveillance testing for all undergraduate students at four timepoints throughout the year, as well as voluntary symptomatic and asymptomatic surveillance testing throughout the year for all students, faculty, and staff. We obtained high quality genome assemblies for a total of 1168 samples representing not only 46.8% (n = 532) of positive samples collected throughout the academic year for University surveillance testing, but also 49.8% (n = 636) of positive samples collected by the nearby local hospital for patients from the surrounding community (hereafter referred to as “Community”) over the same time period. We used these genome sequences to evaluate and compare transmission dynamics within and between the University and the Community by identifying introductions of new viral lineages into the study region and tracing the transmission of those lineages over time. We predicted the University population would have a greater number of introductions of new lineages, and more outbreaks within those lineages, than the Community due to higher rates of travel and more congregate settings for the University population. We also predicted that new introductions and outbreaks would increase following holiday breaks for both populations due to increased travel during these breaks. Finally, we predicted that transmission between the University and the Community would be rare due to social segregation of these populations and mitigation measures employed by both populations.

## Methods

### Study system

**Figure 1:**
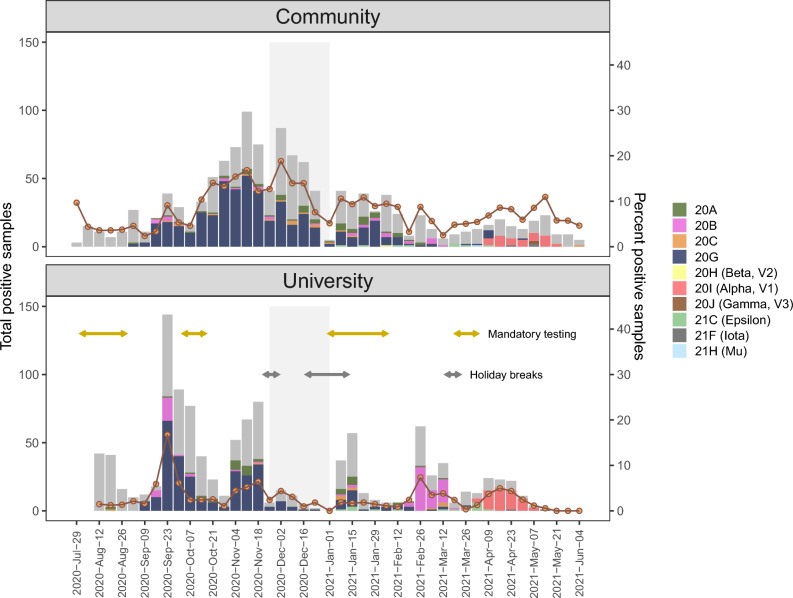
Epidemic curves of SARS-CoV-2 for the local community (top) and the University of Idaho students, staff, and faculty (bottom) during the 2020–2021 academic year. Grey bars indicate the total number of positive cases, and colored bars indicate the numbers of SARS-CoV-2 genomes assembled, colored by clade. Orange line indicates the percent positive samples, calculated as the total number of positive tests divided by the total number of tests performed. Light grey shaded region indicates a period of online-only instruction when fewer students were on campus. Double-sided arrows indicate mandatory SARS-CoV-2 testing periods for undergraduates, and University holiday breaks.

The University is a public land-grant research institution with undergraduate and graduate students located in the city of Moscow in Latah County, Idaho. During the 2020–2021 academic year, the University had approximately 9300 undergraduate students, 2700 graduate students, 900 faculty, and 1500 staff. The University held in-person classes and employed a suite of COVID-19 mitigation strategies including mandatory and voluntary SARS-CoV-2 testing (Table 1, Fig. 1; described further below), mandatory quarantine following a positive SARS-CoV-2 test, masking for indoor on-campus settings except for private workspaces or residential units, physical distancing in classrooms, and hand-sanitizing stations. In addition, courses were held online-only after the Fall break (starting Nov 29) until the start of the Spring semester, during which time students were encouraged to live off-campus at their permanent residences (Table 1, Fig. 1). Approximately 3161 University students lived in on-campus housing during the academic year, including 1603 living in 24 congregate residences of sororities and fraternities.

**Table 1:** Calendar for the University of Idaho for the 2020–2021 academic year, including the start and end dates for semesters, holiday breaks, online-only instruction, mandatory testing periods for undergraduate students, and asymptomatic surveillance testing periods for faculty and staff.

Event	Motivation	Start Date	End Date
Mandatory testing (undergrads)	After summer break	2020-08-01	2020-08-28
Fall semester		2020–08-24	2020-12-18
Asymptomatic surveillance (staff)		2020-09-11	2020-11-20
Mandatory testing (undergrads)	After a spike in cases	2020-10-03	2020-10-16
Fall recess		2020-11-23	2020-11-29
Online-only instruction		2020-11-30	2020-12-17
Winter break		2020-12-18	2021-01-12
Spring semester		2021-01-13	2021-05-14
Mandatory testing (undergrads)	After winter break	2021-01-01	2021-02-05
Asymptomatic surveillance (staff)		2021-02-06	2021-03-19
Spring break		2021-03-15	2021-03-21
Mandatory testing (undergrads)	After spring break	2021-03-20	2021-04-02
Asymptomatic surveillance (staff)		2021-04-03	2021-05-14

The city of Moscow is a rural town with a population size of approximately 26,000 and is located within Latah County, with a population size of approximately 40,000. Throughout the 2020–2021 academic year, the city of Moscow required masking and social distancing in public spaces, and gatherings were restricted to fewer than 10 people. Vaccines became locally available in Moscow in early January for high-risk adults, and in late March for all adults.

SARS-CoV-2 testing for the University during the 2020–2021 academic year was provided by Gritman Medical Center in Moscow (located less than one mile from the University campus), in partnership with the University of Idaho Institute for Interdisciplinary Data Sciences (IIDS) Genomics and Bioinformatics Resources Core (GBRC). Testing was performed using reverse-transcription polymerase chain reaction (RT-PCR, described further below), and turn-around time for patients to receive test results was approximately 4–7 days in early August, 2–3 days from mid-August through October, and 12–36 h for the remainder of the study period. Both symptomatic and asymptomatic testing were provided to all University students and staff free of charge. Mandatory testing for all undergraduate students occurred during four time periods, including the weeks prior to the beginning of classes at the start of the Fall semester, the weeks immediately following two holiday breaks (Winter break and Spring break), and one time period spurred by a large spike in cases in mid-September (Table 1, Fig. 1). In addition, mandatory testing for all undergraduate athletes occurred every Tuesday throughout the academic year. Voluntary asymptomatic surveillance was also performed for all staff and students over three time periods (11 September–20 November, 6 February–19 March, 3 April–14 May)(Table 1). Staff and students received email invitations to voluntarily participate in the weekly asymptomatic surveillance, with individuals randomized so that everyone received one invitation per semester. Staff and students were also invited by campus-wide email to be voluntarily tested prior to any travel throughout the semester.

Gritman Medical Center also provided SARS-CoV-2 testing for the Community during the 2020–2021 academic year. This testing targeted patients who were symptomatic or had been exposed to individuals who tested positive, pre-operative patients, and individuals preparing for domestic or international travel.

### Sample collection

This study includes clinical samples collected at Gritman Medical Center over approximately 9 months from 18 August 2020 to 4 June 2021. This time period began shortly before in-person classes began at the University on 24 August and ended shortly after final exams ended for the spring semester on 14 May (Table 1). Samples were collected using nasopharyngeal swabs and stored in 0.8–0.9% saline and viral transport medium (VTM). RNA was extracted on a KingFisher Flex 96 with a Deep Well head using the MagMaxTM Viral/Pathogen II (MVP II) Nucleic Acid Isolation Kits, which also inactivates samples (Thermo Fisher Scientific, Waltham, MA, USA). Diagnostic RT-PCR testing was performed using the Thermo Fisher Scientific TaqPath COVID-19 Multiplex Diagnostic Solution method, which targets three markers on the genes that encode the ORF1ab, spike (S), and nucleocapsid (N) proteins of the SARS-CoV-2 genome. Results were interpreted using Thermo Fisher Scientific COVID-19 Interpretive Software. A sample was considered positive for SARS-CoV-2 if at least two of the three markers tested positive and would be re-tested if only one marker tested positive. RNA extractions were stored at − 80 °C for approximately 7–16 months prior to whole genome sequencing (described below). Metadata available for each sample included population source (University or Community), age, sex, and sample collection week. University samples were defined as those for which the University had paid for the SARS-CoV-2 test, and all other samples were defined as Community samples. To protect patient confidentiality, patients and samples were deidentified by assigning unique identification codes unrelated to the codes used at Gritman Medical Center. In addition, each sample was assigned to a collection week rather than a collection date, with the first week of each year starting on the first day of the year. The last week of 2020 was only 2 days, and so all samples collected during those days were assigned to the first day of the previous week.

During the study period, testing for a subset of samples collected at Gritman Medical Center was conducted using alternative RT-PCR methods to those described above, including the FilmArray Torch Respiratory Panel 2.1 (BioFire Diagnostics, Salt Lake City, UT, USA), the BD MAX 445003-01 (Becton, Dickinson and Company, Franklin Lakes, NJ, USA), or by outsourced companies. Positive samples identified using these alternative methods were not sequenced for this study because Gritman Medical Center did not store these samples after RT-PCR testing was completed. However, these samples are included in the total weekly counts and positivity rate calculations for COVID-19 cases for both the University and Community (Fig. 1, Fig. S1). These alternative methods were primarily used during the first mandatory University surveillance testing period, which occurred at the beginning of the academic year before the setup of the University testing lab was completed (mid-August to early September; Table 1, Fig. 1), and to a lesser extent throughout the remainder of the academic year.

### Sequencing, assembly, quality control

Whole genome sequencing was conducted for 1966 SARS-CoV-2-positive samples. Library prep was conducted using the HiFiViral SARS-CoV-2 kit (PacBio, Menlo Park, CA, USA), which uses approximately 1000 molecular inversion probes to tile the SARS-CoV-2 genome, with each base of the genome covered by an average of 22 probes. We performed library prep in 96-well plates containing 94 samples and two negative controls, following manufacturer protocols except that library concentrations were normalized prior to sequencing. Libraries were sequenced using Single Molecule, Real-Time (SMRT) sequencing on a PacBio Sequel II, with one plate of samples sequenced per SMRT cell. Library prep and sequencing were performed at the University of Idaho IIDS GBRC.

Sequence reads were analyzed using several applications in the PacBio SMRT Link v.10.2 workflow manager. First, sequencing error was corrected using the Circular Consensus Sequencing Analysis application, which generates highly accurate sequence reads^23^. Next, error-corrected sequence reads were demultiplexed by sample with the Demultiplex Barcodes application in SMRT Link. Genome assembly was then conducted for each sample using the HiFiViral SARS-CoV-2 Analysis application in SMRT Link. This application aligns sequence reads to the Wuhan reference genome (NCBI Reference Sequence NC_045512.2), identifies variants, and then generates a consensus sequence for each sample by injecting variants into the reference genome^24^. We used default threshold values for variant-calling, including a minimum depth of 4 reads and a minimum variant frequency of 0.5. The HiFiViral SARS-CoV-2 Analysis application also flags assemblies exhibiting evidence for the presence of multiple different strains (“multistrain”). A multistrain designation could result from coinfection, contamination, or poor sample quality. We conservatively removed any assemblies that had received a multistrain designation from all subsequent analyses. Remaining assemblies were analyzed using Nextclade v.1.10.0^25^ to evaluate assembly quality, assign a Nextstrain clade, and identify clades designated as variants of concern (VOC) or variants of interest (VOI) by the World Health Organization and the U.S. Centers for Disease Control and Prevention^26^. Assemblies to which Nextclade assigned an overall quality status of “bad” were removed from subsequent analyses. Pango lineages were identified using the UShER mode in Pangolin v.3.1.17^27^ with pangoLEARN 2021-12-06 and pango-designation v.1.2.105.

After removing samples with poor genome assembly quality, we identified any remaining samples which had been collected from the same individual at different timepoints, and compared the similarity of the assembly sequences for these samples based on strain classifications (Pango and Nextstrain classifications) and visual inspection of sequence alignments generated using MUSCLE v.3.8.425^28^ in Geneious v.11.1.5 (Auckland, New Zealand). A total of nine individuals had been sampled twice within a 2-week period, and one individual had been sampled twice approximately 5 months apart. We found that all pairs of samples collected from the same individual within 2 weeks had identical or nearly identical sequences (seven pairs had no nucleotide mismatches, one pair had one mismatch, one pair had two mismatches), indicating that the same variant had been sampled twice for these individuals. Thus, we removed all except one sample for each of these duplicates prior to subsequent analyses. In contrast, the two samples collected from the same individual approximately 5 months apart had 25 nucleotide mismatches and different strain classifications (Nextstrain clades 20G and 21C; Pango lineage B.1.2 and B.1.427), indicating two separate infections for this individual, and thus we retained both sequences for subsequent analyses.

After quality filtering and removal of duplicate samples, all assemblies and associated metadata were submitted to GISAID and NCBI GenBank (BioProject PRJNA788555, Table S1).

### Phylogenetic analyses

We used the Nextstrain ncov workflow v.3.0.6^29^ (https://github.com/nextstrain/ncov/) to build a phylogeny containing our genome assemblies and a contextual subset of assemblies from other locations in Idaho, other states in the USA, other countries in North America, and other countries outside of North America (Tables S2, S3). Within the Nextstrain workflow, we used Augur v.13.1.2^30^ to generate the contextual subset of assemblies by subsampling the GISAID database to obtain a maximum of 1000 assemblies each from Idaho (grouped by year and month), the USA (grouped by state, year, month), North America outside of the USA (grouped by country, year, month), and all countries outside of North America (grouped by country, year, month). All subsampling occurred evenly across groups and restricted samples to dates earlier than 01 July 2021, and sample selection was prioritized based on genetic similarity to the assemblies we generated for this study. Augur was then used to align all assemblies with Nextalign v.1.10.2^25^, and to generate a maximum likelihood phylogeny with IQ-TREE v.2.2.0-beta^31^ as well as a temporally resolved phylogeny using TreeTime v.0.8.6^32^. The temporally resolved phylogeny was visualized using auspice.us (a web application of Nextstrain). The total number of assemblies in the phylogeny was 4118, including the 1168 assemblies generated for this study, as well as 963 assemblies from Idaho, 829 from other USA states, 342 from North American countries outside the USA, and 816 samples from countries outside North America (Tables S2, S3).

We identified introductions of new strains into the University and Community populations using two different methods. First, we used the “introduce” option of matUtils^33,34^ in UShER^35^. For this analysis, we used the publicly available SARS-CoV-2 global phylogeny^33^ created using UShER. This phylogeny uses publicly available SARS-CoV-2 sequences without any down-sampling and is updated daily. At the time of download (20 April 2022), the phylogeny had a total of 4,792,241 sequences, including our genome assemblies. We interrogated the UShER phylogeny using the “matutils introduce” option to identify introductions of new strains into our study populations, as well as introductions of new strains into each US state. We then used these results to identify all sampled descendants for each introduction, which we refer to as the “post-introduction clade” for each introduction, as well as the collection dates and population sources for those sampled descendants. We defined the introduction date as the first date that we detected a descendant of the introduction; this represents an upper bound on the true introduction date into our study populations, since the strain could have been initially introduced at an earlier date by an unsampled individual. We defined the end date for the circulation of introduction descendants in our study populations as the last date that we detected a descendant of the introduction; this represents a lower bound on the true end date, since the lineage could have continued spreading in unsampled individuals. We defined the initial recipient population (University or Community) of each introduction as the population source of the first detected descendant of the introduction. To evaluate the relative contributions of introductions from each US state to our study populations, we calculated log-fold enrichment for introduction rates from each state; this log-fold enrichment procedure accounts for variation in statistical power expected for detecting introduction source locations according to the number of sequences available from each location.

The second method we used to identify introductions of new strains into the University and Community was a discrete trait analysis (DTA) within TreeTime, as implemented using the “augur traits” subcommand within Augur. This method uses a temporally resolved phylogeny to infer the population sources of internal nodes, which represent unsampled ancestral viruses. These inferences can then be used to identify between-population transmission events, defined as branches within the phylogeny for which the parent and child node differ in population source. Our input for the DTA was the phylogeny generated using TreeTime as described above, with the population source for each of our assemblies defined as either University or Community, and for all other contextual assemblies defined as “outside”. The output phylogeny from this analysis included inferred population sources for internal nodes, and we analyzed this phylogeny using the R package ape^36^ in R v.4.1.0^37^ to identify introductions, defined as branches for which the parent node population was “outside” and the child node population was University or Community. We defined the date of each introduction as the date of the child node, which represents an upper bound on the true introduction date. We first performed this analysis defining introductions as all branches that met these criteria, and then only as branches for which the confidence level of population assignment was ≥ 80% for both the parent and child nodes.

We also used the DTA results to investigate viral transmission between and within our focal study populations (Community and University) over time. For this analysis, we used the same phylogeny generated using DTA as described above. To identify between-population transmissions, we used the R package ape to identify all branches for which the child node population source was University and the parent node population source was Community, or vice versa. To identify within-population transmissions, we identified all branches for which the child node population source was University or Community, and the parent node was the same as the child node.

To identify transmission clusters representing large outbreaks, we identified polytomies (i.e., groups of samples with identical sequences) in the Nextstrain maximum likelihood phylogeny using the di2multi function in the R package ape. We characterized the timeframe and composition of each polytomy based on the earliest and latest collection dates of samples from each polytomy, and the number and proportion of individuals from the two populations in each polytomy (University or Community). We defined large polytomies (hereafter “outbreak clusters”) as those with ten or more individuals. For outbreak clusters dominated by University or Community samples (defined as having ≥ 60% of samples originating from one of the two populations), we used Welch’s t-tests to compare the length and size of University-dominated versus Community-dominated outbreaks.

### Ethical approval

Use of SARS-CoV-2-positive clinical samples from Gritman Medical Center and associated metadata (population source, collection week, age, sex) was approved by the Gritman Medical Center HIPAA Compliance Officer, and was determined to not be human subjects research by the University of Idaho Institutional Review Board (IRB) because samples were deidentified by Gritman Medical Center. In addition, all methods were carried out in accordance with relevant guidelines and regulations. The experimental protocols were approved by the University of Idaho IRB and all subjects gave informed consent during sample collection by Gritman Medical Center.

## Results

The total number of SARS-CoV-2 tests performed for Gritman Medical Center during the study period was 56,443, of which 2431 tests were positive (Fig. S1). Of these positive samples, 1966 samples were used for viral genome sequencing in this study. These represented all positive samples that were identified using the TaqPath assay (rather than alternative RT-PCR methods, as described above) and constituted 80.9% of the total number of positive samples collected at Gritman Medical Center during the study period. After quality filtering and duplicate removal, the total number of genome assemblies was 1168 (i.e., 59.4% of samples that were sequenced), including 532 samples from the University and 636 from the Community (Fig. 1, Table S1). These samples represent 46.8% of the total positive University samples and 49.8% of the total positive Community samples collected at Gritman Medical Center during the study period. The weekly proportion of total positive samples with successful genome assemblies was relatively consistent across the study period, except for the first undergraduate general surveillance testing period (mid-August to early September), during which time lower proportions of positive samples were sequenced (Fig. 1). This period had lower sequencing rates because most SARS-CoV-2 testing was outsourced during this time, and sequencing was not performed for any outsourced samples.

For the successful genome assemblies, samples from the University population had a lower median and variance in patient age than the Community, as expected for a population comprised primarily of undergraduate students (University median age = 20 years, standard deviation = 6.77; Community median age = 33 years, standard deviation = 19.3) (Fig. S2). Females and males were approximately equally represented within each population (263 females and 269 males for University, 318 females and 318 males for Community). Samples that failed genome assembly quality control had higher Ct values, consistent with an increased failure rate for samples with low viral load (median Ct = 29.6 for the N gene for samples that failed, 19.2 for samples that passed, Fig. S3).

### Temporal variation in positivity rates

Temporal variation in positivity rate differed for the University versus Community (Fig. 1). The University had four distinct, relatively short-lived waves characterized by rapid increases and decreases in positivity rates. In contrast, the Community had two waves, with the first wave lasting longer than any of the University waves, and both Community waves were generally aligned temporally with waves in most adjacent counties (Fig. S4). Three of the four University waves occurred during Community waves and had similar proportions of Nextstrain clades as those present in the Community at that time (Fig. 1, Fig. S5). These three University waves peaked in mid-September (near the beginning of the academic year), mid-November, and mid-April (a wave of the Alpha variant, known to be more infectious than previous variants). In contrast, a fourth University wave occurred in the time between the two Community waves. This wave lasted from mid-February to mid-March and involved a clade that was relatively rare throughout the rest of the study period (clade 20B) for both populations.

The University reported that the first wave in mid-September was strongly dominated by students living in the congregate residences of sororities and fraternities, and 14 of the 24 residences were placed in quarantine by the University. The February–March wave also included students in sororities and fraternities, but to a lesser extent than the first wave. The remaining waves were not reported by the University to be dominated by certain demographic groups or housing condition. Comparison of temporal variation in positivity rates for females versus males showed little difference overall between the sexes, but the mid-September University wave had more than twice as many females as males, and the February–March University wave had more than twice as many males as females (Fig. S6).

Spring break (2021-03-15 to 2021-03-21) was the only holiday break that was followed by an increase in positivity rate for both the University and Community, and these increases were driven by the more infectious Alpha variant, which was first detected in our dataset immediately following Spring break. The Community also experienced an increase in positivity rate following the Fall break. In contrast, the Winter break was not followed by increases in positivity rates for either population, even though this break was followed by an increase in introductions of new viral lineages (described further below). Notably, however, the Community positivity rate was relatively high both before and after the Winter break (~ 10%).

**Figure 2:**
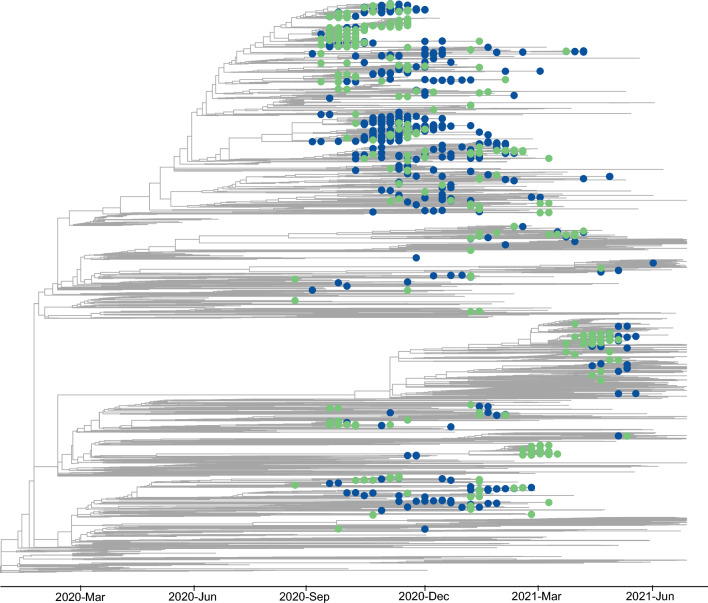
Time-calibrated maximum likelihood phylogeny of 4118 SARS-CoV-2 genomes, including genomes sequenced for this study from the University of Idaho students, staff, and faculty (green circles) and from the local community (blue circles). Other global samples are indicated by branch tips without circles. An interactive version of this phylogeny is available at https://nextstrain.org/community/narratives/kimandrews/UofISARSCoV2.

A total of ten Nextstrain clades were present in our dataset (Figs. 1, 2). For both the University and Community, the dominant clade for most of the study period was 20G; this clade dominated both populations from the beginning of the study period until mid-February. As described above, the University experienced a wave of clade 20B from February–March, followed by a wave of the Alpha variant in both the Community and University from April–May, shortly after the first introduction of the Alpha variant in late March. Five additional VOC or VOI were detected in our dataset, but at relatively small numbers (n = 24 for Epsilon, n < 5 each for Beta, Gamma, Iota, Mu) when compared to the Alpha variant (n = 100).

### Introductions

**Figure 3:**
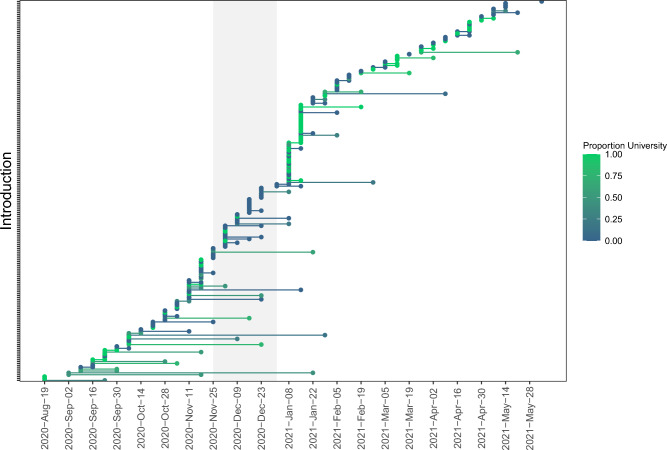
Start and end dates for SARS-CoV-2 post-introduction clades identified by analysis of the UShER phylogeny, with the start date defined as the date that descendants of the introduction were first sampled, and the end date defined as the last date that any descendants of the introduction were sampled. Introductions are color-coded based on the proportion of sampled individuals in the post-introduction clade that were from the University (green) or the local Community (blue). Light grey shaded region indicates a period of online-only instruction when fewer students were on campus. Introductions that were variants of concern or variants of interest are indicated in Figure S10.

**Figure 4:**
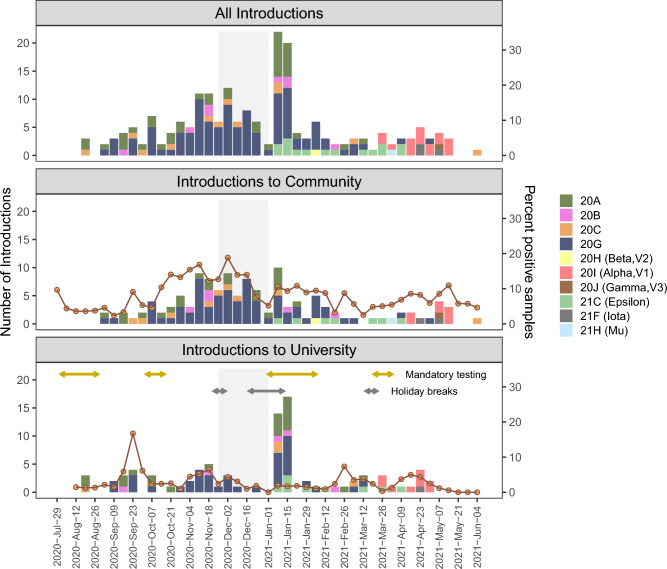
Counts of new introductions of SARS-CoV-2 variants into the local community and the University of Idaho during the 2020–2021 academic year identified by analysis of the UShER phylogeny, with introduction dates defined as the date that descendants of the introduction were first sampled. The top panel shows counts of all new introductions, and the bottom two panels show counts of new introductions into the Community (middle) or University (bottom). Introductions are color-coded by clade. Orange line indicates the percent positive samples over time for each recipient population. Light grey shaded region indicates a period of online-only instruction when fewer students were on campus. Double-sided arrows indicate mandatory SARS-CoV-2 testing periods for undergraduates, and University holiday breaks.

Analyses identifying introductions of new viral lineages produced similar results for the approaches using the UShER phylogeny (Figs. 3, 4, Figs. S7–S10, Tables S4, S5) and the DTA (Fig. S11, Table S6). The UShER approach identified a total of 202 introductions of new viral lineages into the study region, and the DTA identified 206 introductions, including 162 high-confidence introductions. Both methods identified more introductions into the Community than the University: UShER identified 125 introductions into Community, 88 into the University, and 11 with an unclear initial recipient population (i.e., the first descendants of the introduction included both Community and University samples from the same date), and DTA identified 131 introductions into the Community and 75 into the University. When including only high-confidence introductions for the DTA, the number detected was 95 into the Community and 67 into the University. For the Community, introductions were more frequent in the Fall semester than the Spring semester, corresponding with high positivity rates during this time (Figs. 3, 4). For the University, a large peak in introductions occurred immediately following Winter break, when positivity rates were low for both populations. These post-Winter break introductions were detected during a period of mandatory undergraduate surveillance testing, and the low positivity rate during this time indicates that these introductions represented a relatively small proportion of the total tests performed.

The UShER analysis of post-introduction clades indicated that more than half of introductions were “phylogenetic singletons” (n = 115 of the 202 introductions), defined as introductions represented in the dataset by only one sample and no additional descendants in the phylogeny (Fig. 3). These singletons indicate introductions that led to minimal transmission within the study region. Notably, most of the introductions identified during the mandatory undergraduate testing period immediately following the Winter break were singletons, indicating limited transmission within the study region, potentially due to the University-mandated quarantine of individuals that tested positive.

The UShER analysis identified a total of 12 “large” post-introduction clades, defined as clades comprised of > 10 descendant samples (Figs. S7–S9). The largest post-introduction clade was comprised of 41.8% of all samples in the study (n = 488 samples), including samples collected over approximately 4 months from early September to mid-January, indicating extended transmission resulting from this introduction. This introduction was first detected early in the study period and may have been initially introduced into the study region prior to the start of our sampling. Other non-singleton introductions detected in our dataset yielded clades ranging in size from 2 to 74 samples (Fig. S7). The Fall semester had longer-lasting post-introduction clades than the Spring semester, with many clades lasting several months, but none lasting beyond the end of the first Community wave in February (Figs. 3, 4).

Of the 12 large post-introduction clades identified by the UShER analysis, four were dominated by samples from the University (i.e., ≥ 60% of samples in the clade were from the University) and five were dominated by samples from the Community, including two clades comprised of 100% Community samples (Fig. S9). The four University-dominated clades lasted between approximately 28 and 56 days, and the five Community-dominated clades lasted between approximately 21 and 114 days. Only one large post-introduction clade was dominated by samples from one sex; this clade included 27 samples, of which 74% were female and 78% were from the University.

Of the 202 introductions detected by the UShER analysis, 37 were introductions of VOCs, including 16 introductions of Alpha, 15 introductions of Epsilon, three introductions of Iota, and one introduction each for Beta, Gamma, and Mu (Fig. S10). One introduction of the Alpha variant resulted in 74 descendants, but all other VOC introductions had fewer than seven descendants.

The highest log-fold enrichment value for introductions to the study region identified by the UShER analysis was for the state of Washington (0.67), with 52 introductions detected (Table S5). The second-highest value was for Idaho (0.40), with only two introductions detected. High log-fold enrichment values indicate large numbers of introductions from a state relative to the total number of sequences available from that state. Thus, the high log-fold enrichment value for Idaho despite a low number of introductions suggests a low total number of sequences available from Idaho, and thus low power to identify Idaho as a source for introductions to the study region. In addition, the source population was indeterminate for a total of 47 introductions into our study region, indicating low power to detect source states for many introductions, likely including Idaho and potentially other states with low sequence availability.

### Transmission between populations

**Figure 5:**
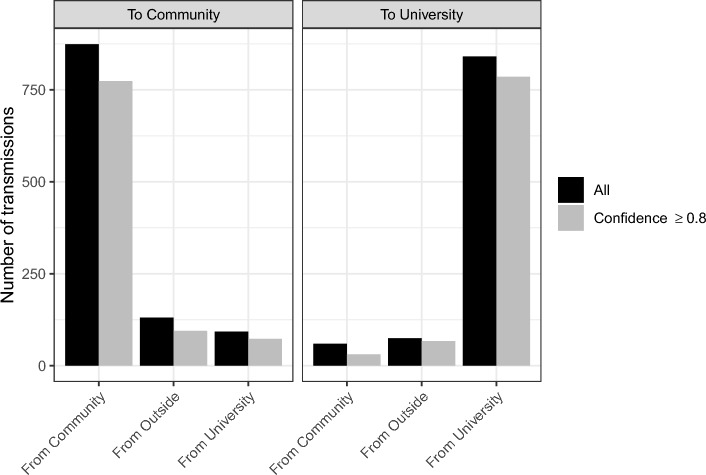
Numbers of between-population transmissions identified by discrete trait analysis, including transmissions into the local community (left panel) or University of Idaho (right panel). Black bars indicate all transmissions identified, and grey bars indicate only high confidence transmissions (population assignment confidence ≥ 0.8 for parent and child nodes). “Outside” = population other than our focal study populations.

The DTA indicated the majority of viral transmissions in our study system occurred within populations rather than between populations, with 79.6% of transmissions into the Community originating from the Community (n = 874 of 1098 total transmissions), and 86.2% of transmissions into the University originating from the University (n = 841 of 976 total transmissions) (Fig. 5, Table S6). Of the remaining transmissions, most were from sources outside our focal study populations, with 11.9% of transmissions into the Community being introductions from outside sources (n = 131 transmissions), and 7.7% of transmissions into the University being introductions from outside sources (n = 75 transmissions) (Fig. 5, Fig. S11, Table S6). For transmissions between our focal study populations, rates were slightly higher from the University to the Community than in the opposite direction, with 8.5% of transmissions to the Community originating from the University (n = 93 transmissions), and 6.1% of transmissions to the University originating from the Community (n = 60 transmissions) (Fig. 5, Fig. S12a, Table S6). When including only transmissions with high confidence levels, these values declined to 7.7% of transmissions to the Community from the University (n = 73 of 942 total transmissions), and 3.5% of transmissions to the University from the Community (n = 31 of 884 total transmissions) (Fig. 5, Fig. S12b, Table S5). A large percentage of between-population transmissions were phylogenetic singletons, indicating limited onward transmission into the recipient population (78.5% of transmissions from University to Community, 76.7% of transmissions from Community to University; for high-confidence transmissions only: 89.0% of transmissions from University to Community, 90.3% of transmissions from Community to University). Most transmissions from the University to Community occurred during peaks in positivity rates for the University, whereas transmission in the opposite direction did not correspond strongly with positivity rate for the Community (Fig. S12).

### Outbreaks

**Figure 6:**
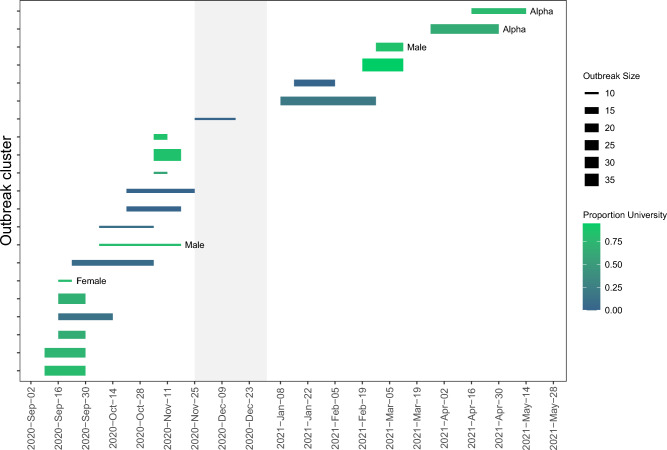
Start and end dates for large SARS-CoV-2 outbreak clusters, color-coded by the proportion of individuals in the cluster from the University (green) and the local community (blue), with thickness of bars representing the numbers of individuals in the cluster. Two outbreak clusters that were the alpha variant are indicated; no other outbreak clusters were variants of concern or variants of interest. Three outbreak clusters that were dominated by one sex are indicated by “Female” or “Male”. Light grey shaded region indicates a period of online-only instruction when fewer students were on campus.

**Figure 7:**
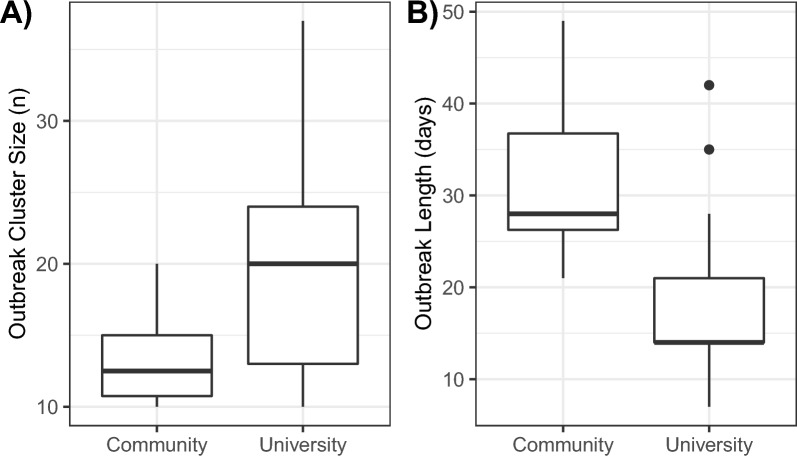
Distributions of sizes (**A**) and lengths (**B**) of SARS-CoV-2 outbreak clusters for the University of Idaho and the local community.

We identified a total of 21 outbreak clusters with ten or more individuals (Figs. 6, 7, Figs. S13–S15). These accounted for 31.3% of all samples (i.e., a total of 365 samples) and included individuals from nine of the 202 post-introduction clades. The largest post-introduction clade in the dataset gave rise to 11 of the 21 outbreak clusters, and all other post-introduction clades gave rise to just one or two clusters. A total of 13 clusters were dominated by the University (i.e., ≥ 60% of samples in the cluster were from the University), and eight clusters were dominated by Community (≥ 80% of samples) (Fig. 6, Fig. S15).

Most outbreak clusters occurred during the Fall semester, with 14 clusters beginning and ending by 2 December, and a large number of those clusters occurred at the start of the academic year, with six clusters beginning within the first month of the Fall semester (Fig. 6). The final two outbreak clusters were the Alpha variant (outbreaks starting in late March and mid-April); both of these clusters were comprised primarily of University samples and originated from the same post-introduction clade (Fig. 6). No other outbreak clusters were VOCs or VOIs. As expected based on population age composition, University-dominated outbreak clusters had lower median and variance in age than Community-dominated outbreak clusters, with median values ranging from 19 to 25.5 across clusters (standard deviation ranging from 0.57 to 12.0), although age ranged up to 64 in one University-dominated cluster (Fig. S15). For Community-dominated outbreak clusters, ages ranged from 6 to 84, median ages ranged from 23 to 44, and standard deviations ranged from 15.5 to 27.8 (Fig. S15). University-dominated outbreak clusters were concentrated at the beginning of the academic year but occurred throughout the study period except for a 3-month period from mid-November to mid-February, including a time period when most students were away from campus from 23 November until 1 January (Fig. 6). In contrast, Community-dominated outbreak clusters occurred throughout the study period except for the last 3 months, from late-February to the end of the study (Fig. 6). University-dominated outbreak clusters had larger numbers of samples (mean = 19.9 individuals for University, 13.3 individuals for Community; *p* = 0.02), but lasted a shorter amount of time (mean = 18.8 days for University, 31.5 days for Community; *p* = 0.01) (Fig. 7). Most outbreak clusters had similar numbers of males and females, but three University clusters were dominated by one sex (one with 100% females, two with ≥ 80% males) (Fig. 6, Fig. S15). The female-dominated cluster occurred near the beginning of the study period and lasted approximately 1 week, and was comprised of 10 individuals (Fig. 6). One of the male-dominated clusters began early in the study, included 10 individuals, and lasted approximately 6 weeks, and the second male-dominated cluster began in late February, included 20 individuals, and lasted approximately 6 weeks (Fig. 6).

Time periods with large numbers of outbreak clusters also had high positivity rates (Figs. 1, 6). The peak of the first University wave corresponded with five University clusters and two Community clusters, and the peaks of the second University wave and the first Community wave occurred at approximately the same time and corresponded with two University clusters and three Community clusters. In contrast, the last two University waves and the last Community wave each corresponded with just two University clusters, indicating that these waves may have been driven by fewer exposure sources (e.g., fewer social gatherings), although contact-tracing data would be needed to identify the number of exposure sources.

## Discussion

Transmission dynamics differed between the University and Community. Whereas the University had four relatively short-lived waves characterized by rapid increases and decreases in cases, the Community had two waves, the first of which lasted a large portion of the study period. Genomic surveillance indicated the University waves were characterized by larger numbers and sizes of outbreak clusters than those in the Community, potentially due to more congregate settings for the University. However, outbreak clusters also lasted for a shorter length of time for the University, potentially resulting from mitigation measures implemented by the University, including a surveillance testing program and quarantine of infected individuals. This result is consistent with a previous study which found that IHE surveillance testing programs influence SARS-CoV-2 transmission dynamics, as evidenced by lower rates of COVID-19 cases and deaths in US counties with IHEs that implemented these programs in Fall 2020^11^.

Genomic surveillance indicated that transmission of SARS-CoV-2 between the University and Community was limited throughout the academic year, consistent with previous studies of other IHEs^5,9^. Only about 8% of detected transmissions into the Community originated from the University, and about 6% of detected transmissions into the University originated from the Community. Also, temporal variation in positivity rate was similar for the Community as for adjacent counties that did not have IHEs, providing evidence that the University had minimal influence on Community dynamics. Most outbreak clusters identified in our dataset were dominated by samples from only one of the two populations (either University or Community), and most of the large post-introduction clades were also dominated by just one population. Furthermore, the third University wave, which was dominated by a clade that was rare in the study system, resulted in minimal transmission into the Community. Nonetheless, some transmission between populations was evident, with many post-introduction clades and outbreak clusters having some samples from both populations, and with clade composition being similar for the two populations throughout most of the study period.

### Characterizing infection waves

As observed for many IHEs in the 2020–2021 academic year, the University had a rapid surge in cases at the beginning of the Fall semester, which was quickly followed by a rapid decrease in cases^1,3^. This surge occurred despite a low positivity rate during mandatory undergraduate testing at the beginning of the Fall semester, and could be caused by a variety of factors including transmission while waiting for test results (approximately 4–7 days turnaround time for the first surveillance effort, whereas turnaround time was < 48 h for most of the remainder of the study period) or false negative tests for individuals recently infected but not yet having detectable levels of SARS-CoV-2. Alternatively, transmission after surveillance testing could have occurred from University faculty or staff, the local community, or other outside sources. Genomic data indicated the first University wave was associated with multiple large outbreak clusters within the University population, suggesting multiple high-transmission sources, such as social gatherings. The University reported this wave was dominated by individuals living in sorority and fraternity houses, and the University took action to place 14 of these houses in quarantine, which was followed by an observed rapid decrease in cases. Overall these results indicate that heightened mitigation measures at the start of the semester would be beneficial, such as decreased turn-around time for test results, quarantine of students until results have been received, and follow-up testing, especially for individuals in high-risk groups such as sororities and fraternities.

The second University wave occurred just prior to the Fall break during peaks in the numbers of new introductions into the study region as well as transmissions from the Community to the University, indicating that travel and between-population transmission could have contributed to the surge in cases. This wave was also associated with multiple outbreak clusters both within the University and within the Community, suggesting multiple high-transmission source events for both populations. The final two University waves occurred in the Spring semester and were each associated with only two outbreak clusters, potentially indicating a smaller number of high-transmission events compared to the first two waves in the Fall semester. The final University wave was driven by the more infectious Alpha variant and occurred simultaneously with an Alpha variant wave in the Community. This was the only University wave that occurred immediately following a holiday break (i.e., Spring break). The University reported no specific demographic groups disproportionately involved in any of the final three University waves.

### Introductions of viral lineages

We identified approximately 200 introductions of new SARS-CoV-2 viral lineages into the study region, with about 1.5 times as many introductions into the Community as the University. The highest numbers of introductions occurred immediately following Winter break in early January, likely resulting from travel to and from the study region during this break. Most of these introductions were detected in University samples collected during the post-Winter break mandatory undergraduate surveillance testing, and most were phylogenetic singletons, indicating limited subsequent transmission into the study populations. Furthermore, positivity rates did not increase following the Winter break for either the University or Community. These results indicate that the post-Winter break asymptomatic surveillance effort was likely effective in stopping transmission of new introductions following holiday travel.

Introductions were also high around the Fall break in mid-November, potentially resulting from holiday travel. In contrast, Spring break was not associated with an increase in the number of introductions. Nonetheless, the more-infectious Alpha variant was introduced immediately after Spring break, and this introduction was quickly followed by a wave of the Alpha variant in both the University and Community. Thus, Spring break travel likely influenced transmission dynamics in our study region despite a relatively small number of introductions following this break, because it increased the probability of introduction for the more-infectious Alpha variant.

We also found that new introductions occurred overall more frequently in the Fall semester than the Spring semester. This result could indicate a higher risk of introductions during the Fall semester, potentially due to higher caseloads across the US during that time period. Notably, many of the viral lineages identified as new introductions early in the Fall semester may have been present in the local community prior to the start of our study.

### Comparison with other IHEs

Transmission dynamics of SARS-CoV-2 at the University shared many features with other IHEs. Along with many other IHEs, the University experienced a surge in cases soon after the start of the Fall 2020 semester^1,3^. Sororities and fraternities were an important source of transmission during this initial surge, consistent with observations at other IHEs^5,12,14^. The timing of the remaining University waves (peaking in November, February, and April) was also similar to that of waves at multiple Massachusetts IHEs, although those IHEs did not have a large wave at the start of the Fall 2020 semester^11^. Many additional IHEs also had a wave of the Alpha variant in the Spring semester, although the timing of this wave varied across IHEs^38,39^. The presence of differences in the timing and duration of infection waves for the University versus the surrounding community was also observed for multiple IHEs in Massachusetts during the 2020–2021 academic year^11^. As in our study system, the Massachusetts IHEs had more short-lived waves than their surrounding communities^11^. In addition, Purdue University had a greater prevalence of VOC in the university population than the surrounding community during the Spring 2021 semester, potentially resulting from greater levels of travel for the university population^39^. In the following academic year (2021–2022), the Omicron variant spread more rapidly through three Massachusetts IHEs than the surrounding community, potentially resulting from differences in social structure and demographics between university and community populations^40^. Our finding of limited transmission from the University to the surrounding community was also similar to findings in other IHEs^5,8,9,12^. Importantly, however, even a small number of transmission events can have serious consequences for vulnerable populations in communities surrounding IHEs^1–4^. Many of the other transmission dynamic features identified in this study cannot be directly compared with other IHEs due to a lack of studies conducting similar types of analyses.

### Limitations

This study had several limitations. Sampling of the University population was more aggressive than the Community due to the mitigation measures implemented by the University, including asymptomatic testing, and this difference could result in increased estimates of proportional representation of the University population for outbreaks, introductions, and between-population transmissions^41^. However, the large sample sizes and relatively balanced spatiotemporal sampling of our study are predicted to reduce this sampling bias^42^. Our comparison of University and Community transmission dynamics could also be influenced by the presence of some overlap between these two populations. For example, the University population in this study includes staff and faculty, many of whom live off campus and interact substantially with the Community population. In addition, some individuals in the University population may have chosen to receive SARS-CoV-2 tests at Gritman Medical Center outside of the University-sponsored testing program, and these individuals would have been classified as belonging to the Community population in this study. Another limitation of this study is the absence of contact tracing data associated with our samples. When combined with genomic surveillance data, contact tracing information can often confidently identify specific outbreak sources, such as specific dormitories, care homes, social events, or classrooms^5,8,12^.

### Future recommendations

This study demonstrates the value of surveillance testing and quarantine for mitigation of COVID-19 outbreaks at the University, and also identifies high-risk time periods and demographic groups for which increased surveillance testing would be valuable. In particular, increased surveillance testing could be valuable for sorority and fraternity members during the first month of the Fall semester, and for all undergraduate students during times of high caseloads in the surrounding community. In addition, our study demonstrated the value of testing all undergraduate students following holiday breaks, to stop the transmission of new variants introduced from holiday travel. Increased testing effort for these time periods and demographic groups could include increasing the numbers of individuals tested and the frequency of testing, decreasing the turn-around time for test results by using rapid tests, wastewater testing^43,44^, and other efforts.

## Conclusions

Surveillance testing and viral genome sequencing for the University of Idaho and the surrounding local community indicated that transmission dynamics differed between these two populations, with the University having a greater number of infection waves, but more short-lived waves. This dynamic could have been driven by the large number of congregate settings that are characteristic of IHEs and that can lead to rapid outbreaks, combined with effective mitigation measures implemented by the University to combat outbreaks. We identified multiple transmission risk factors for the University that varied in importance across the academic calendar, including congregate settings such as sorority and fraternity events and residences, leading to within-population transmission; high caseloads in the local community, leading to transmission into the University population; and holiday travel, leading to new introductions into the study region, including the introduction of a more infectious variant (i.e., the Alpha variant). Knowledge of these risk factors, and how they vary over time, can help the University of Idaho and other IHEs develop, refine, and prioritize effective mitigation measures against SARS-CoV-2 and other pathogens.

## Supplementary Information

Supplementary Figures.

Supplementary Tables.

## Data Availability

Genome assemblies and associated metadata are available on GISAID and NCBI GenBank (BioProject PRJNA788555, Table S1). Newick-format phylogenetic tree files and custom scripts used for data analysis are provided at https://github.com/kimandrews/UofISARSCoV2. An interactive phylogeny is available at https://nextstrain.org/community/narratives/kimandrews/UofISARSCoV2.
