# Author Correction: Genomic surveillance identifies potential risk factors for SARS-CoV-2 transmission at a mid-sized university in a small rural town

**DOI:** 10.1038/s41598-023-36764-3

**Published:** 2023-06-19

**Authors:** Kimberly R. Andrews, Daniel D. New, Digpal S. Gour, Kane Francetich, Scott A. Minnich, Barrie D. Robison, Carolyn J. Hovde

**Affiliations:** 1 Institute for Interdisciplinary Data Sciences, University of Idaho, Moscow, ID 83844 USA; 2 Gritman Medical Center, Moscow, ID 83843 USA; 3 Department of Animal, Veterinary and Food Science, University of Idaho, Moscow, ID 83844 USA

Correction to: *Scientific Reports* 10.1038/s41598-023-34625-7, published online 16 May 2023

The original version of this Article contained an error in the Acknowledgements section.

“Financial support for this study was provided by NIH Surveillance NOSI NOT-GM-21-031 and by the University of Idaho IIDS Genomics and Bioinformatics Resources Core, which is supported in part by NIH COBRE Phase III Grant P30GM103324.”

now reads:

“The project described was supported by an Institutional Development Award (IDeA) from the National Institute of General Medical Sciences of the National Institutes of Health under Grant #P20GM103408-21S1 and the University of Idaho IIDS Genomics and Bioinformatics Resources Core, which is supported in part by NIH COBRE Phase III Grant P30GM103324.”

The original Article has been corrected.

