# Supplementary material for: Genomic surveillance identifies potential risk factors for SARS-CoV-2 transmission at a mid-sized university in a small rural town
Source: Sci Rep. 2023 May 16;13:7902. doi: 10.1038/s41598-023-34625-7 (PMC10185956; doi:10.1038/s41598-023-34625-7)
Supplement: Supplementary file 1 — Supplementary Figures. [file 41598_2023_34625_MOESM1_ESM.docx]

**Genomic surveillance identifies potential risk factors for SARS-CoV-2 transmission at a mid-sized university in a small rural town**

Kimberly R. Andrews*^1^, Daniel D. New^1^, Digpal S. Gour^1^, Kane Francetich^2^, Scott A. Minnich^3^, Barrie D. Robison^1^, Carolyn J. Hovde^3^

^1^Institute for Interdisciplinary Data Sciences, University of Idaho, Moscow, ID 83844, USA

^2^Gritman Medical Center, Moscow, ID 83843, USA

^3^Department of Animal, Veterinary and Food Science, University of Idaho, Moscow, ID 83844, USA

Corresponding author: Kimberly R. Andrews, kimberlya@uidaho.edu

**Supplementary Tables**

All supplementary tables and table legends are provided in one Excel file, with each supplementary table in a separate worksheet.

**Supplementary Figures**


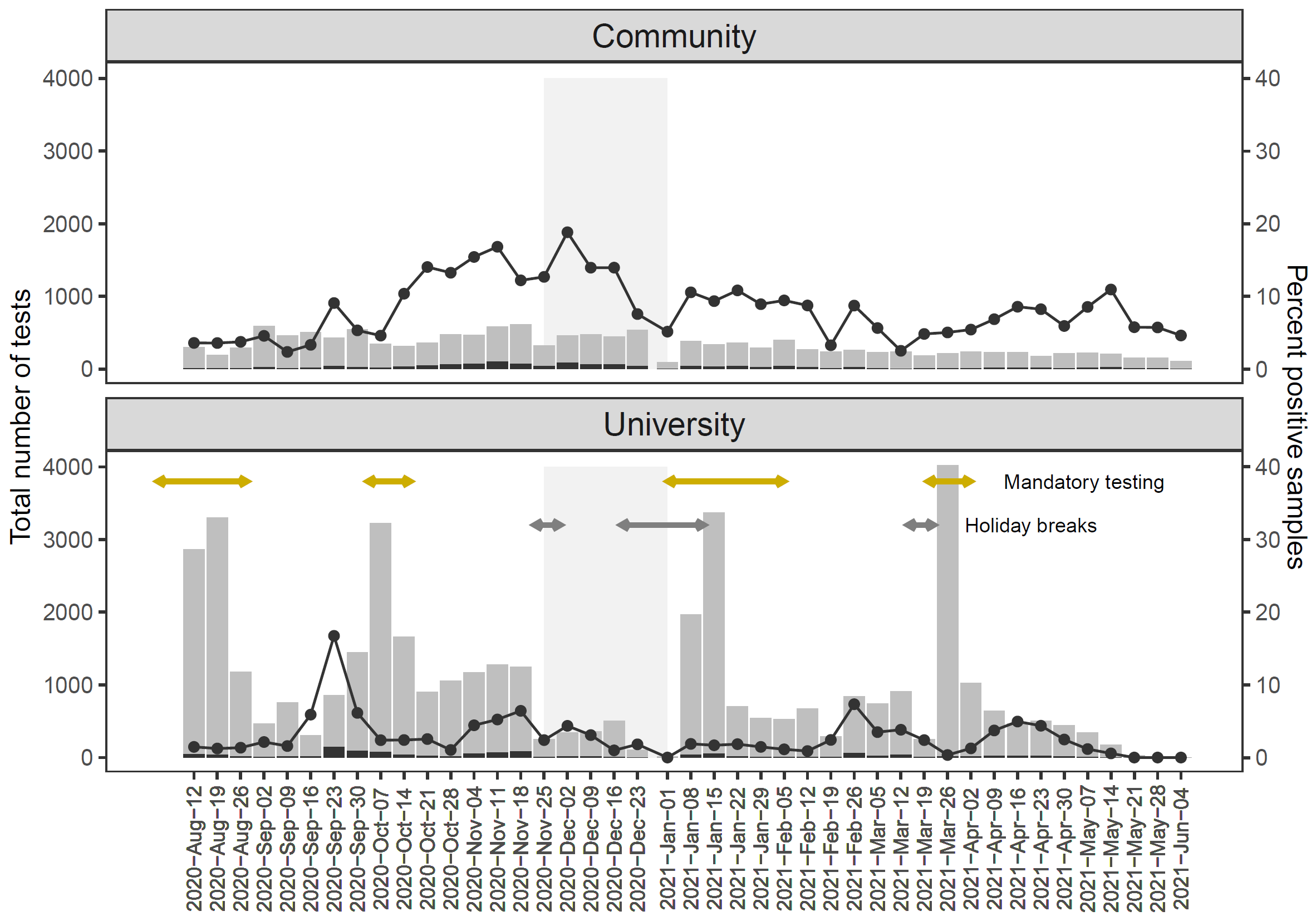


Figure S1. Total numbers of SARS-CoV-2 tests (light gray bars), total numbers of positive tests (black bars), and positivity rates (black lines) for samples collected at Gritman Medical Center for the local community (top) and University of Idaho students, staff, and faculty (bottom) during the 2020-2021 academic year. Light grey shaded region indicates a period of online-only instruction when fewer students were on campus. Double-sided arrows indicate mandatory SARS-CoV-2 testing periods for undergraduates, and University holiday breaks.


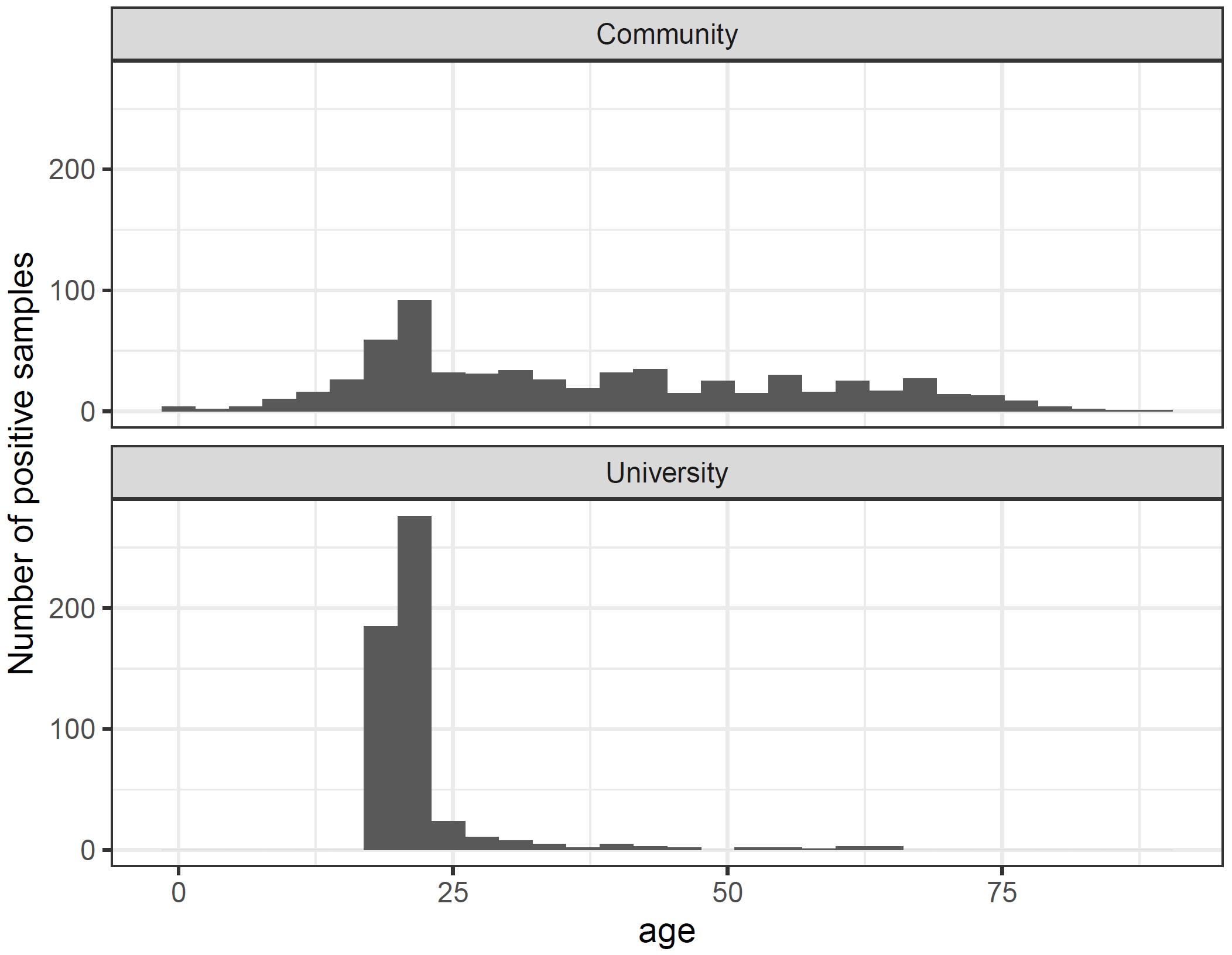


Figures S2. Distributions of patient ages for SARS-CoV-2 genomes from the local community (top) and the University of Idaho (bottom).


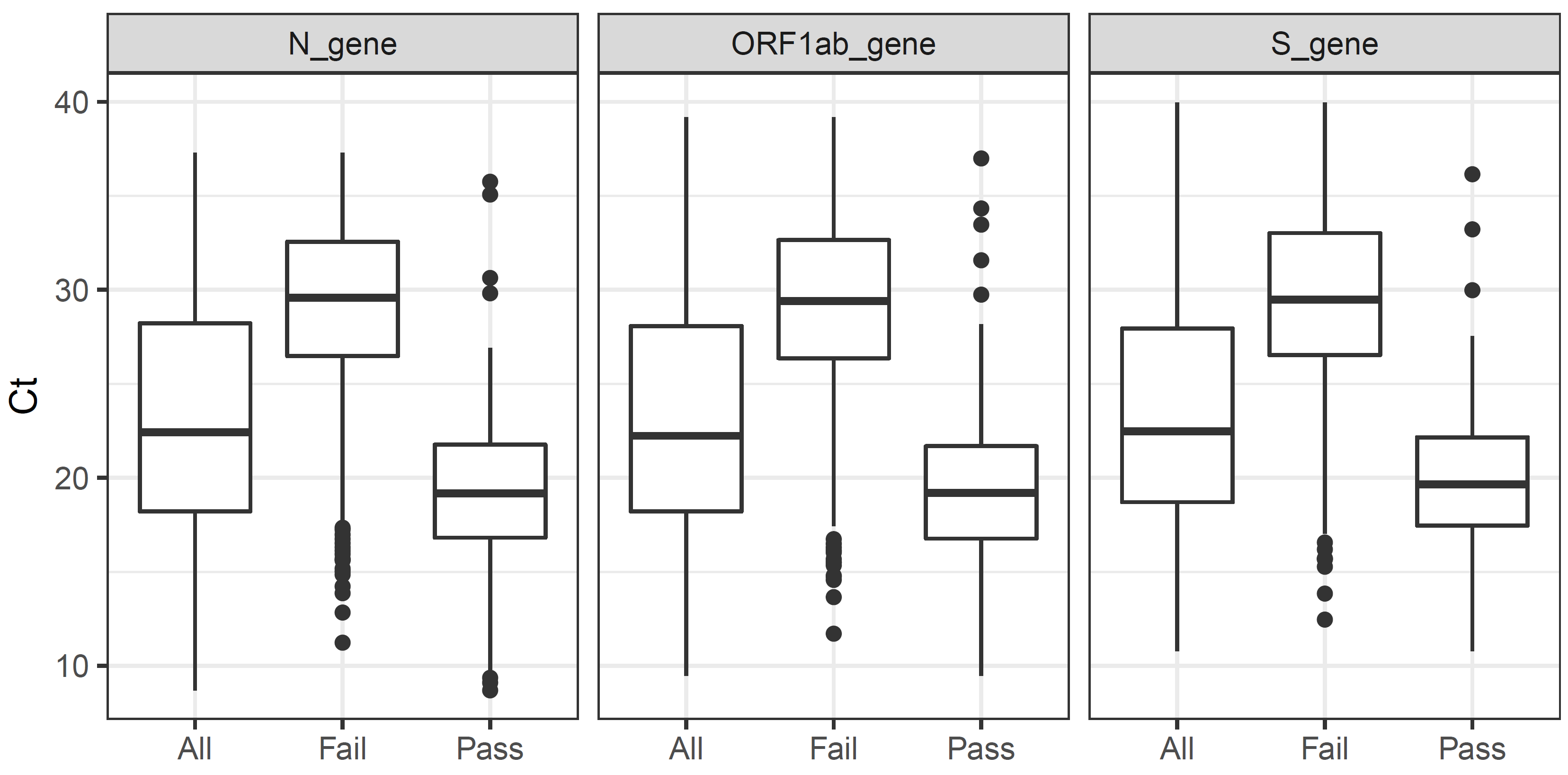


Figure S3. Distributions of RT-PCR Ct scores for SARS-CoV-2 samples collected from the University of Idaho and the local community. Distributions are plotted separately for all samples combined (“All”), for the subset of samples that failed genome assembly quality control (“Fail”), and for the subset of samples that passed quality control (“Pass”). Scores were generated using the ThermoFisher TaqPath COVID-19 Multiplex Diagnostic Solution method, which targets three gene regions (N protein, ORF1ab, S protein).


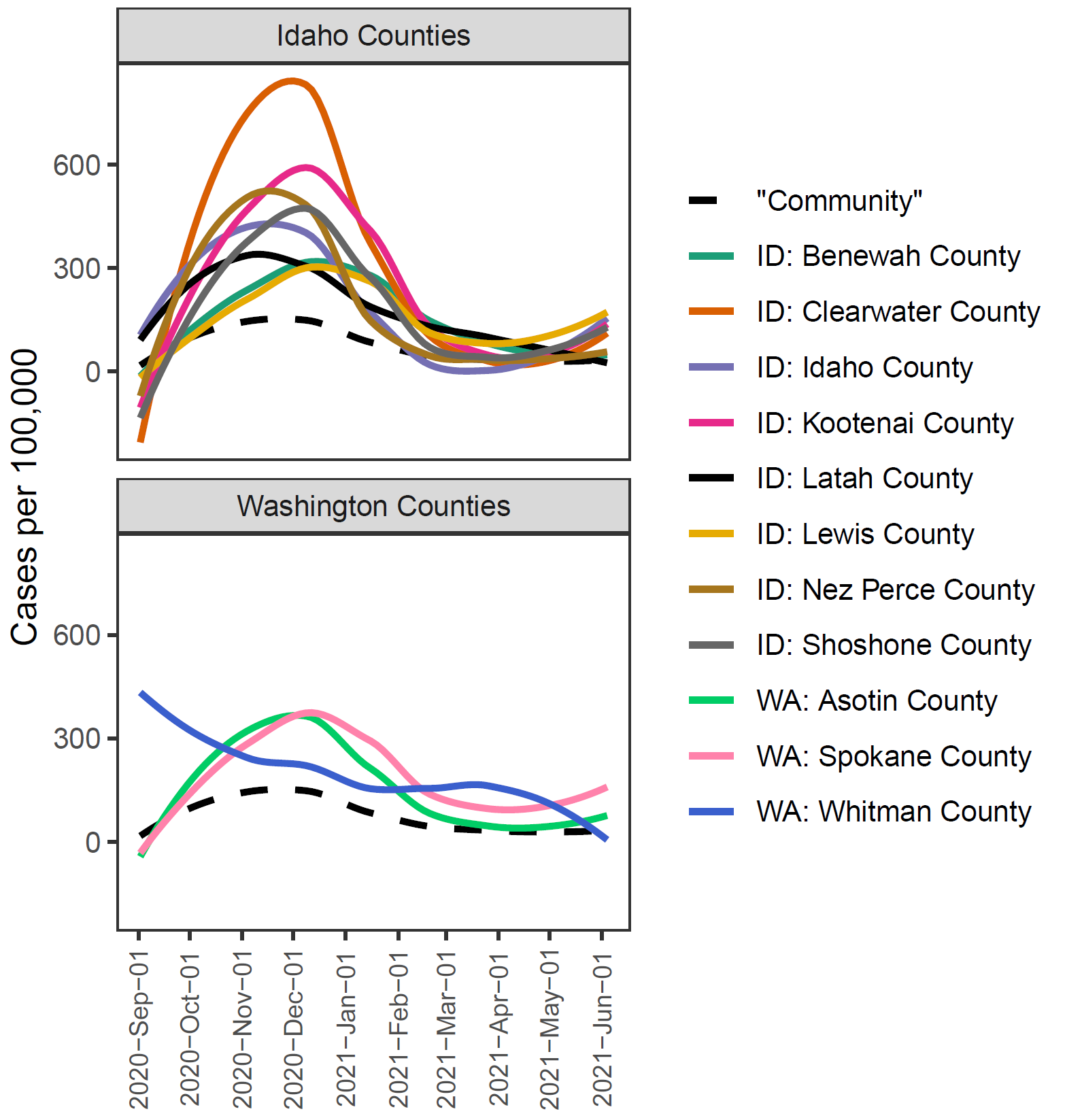


Figure S4. Comparing temporal variation in COVID-19 cases for the local community (“Community”, dashed black line) versus the entire local county (Latah County, solid black line) and neighboring counties in Idaho and Washington. Results for “Community” are shown in both the Idaho and Washington plots for comparative purposes. The nearest counties to Latah that have metropolitan areas are Kootenai (~170,000) and Spokane (~540,000), and all other counties have population sizes <85,000. Note that Whitman County is home to an IHE (Washington State University) which had the same Fall 2020 semester start date as the University of Idaho (24 August). Daily case numbers were downloaded on 28 April 2022 from https://usafacts.org/visualizations/coronavirus-covid-19-spread-map.


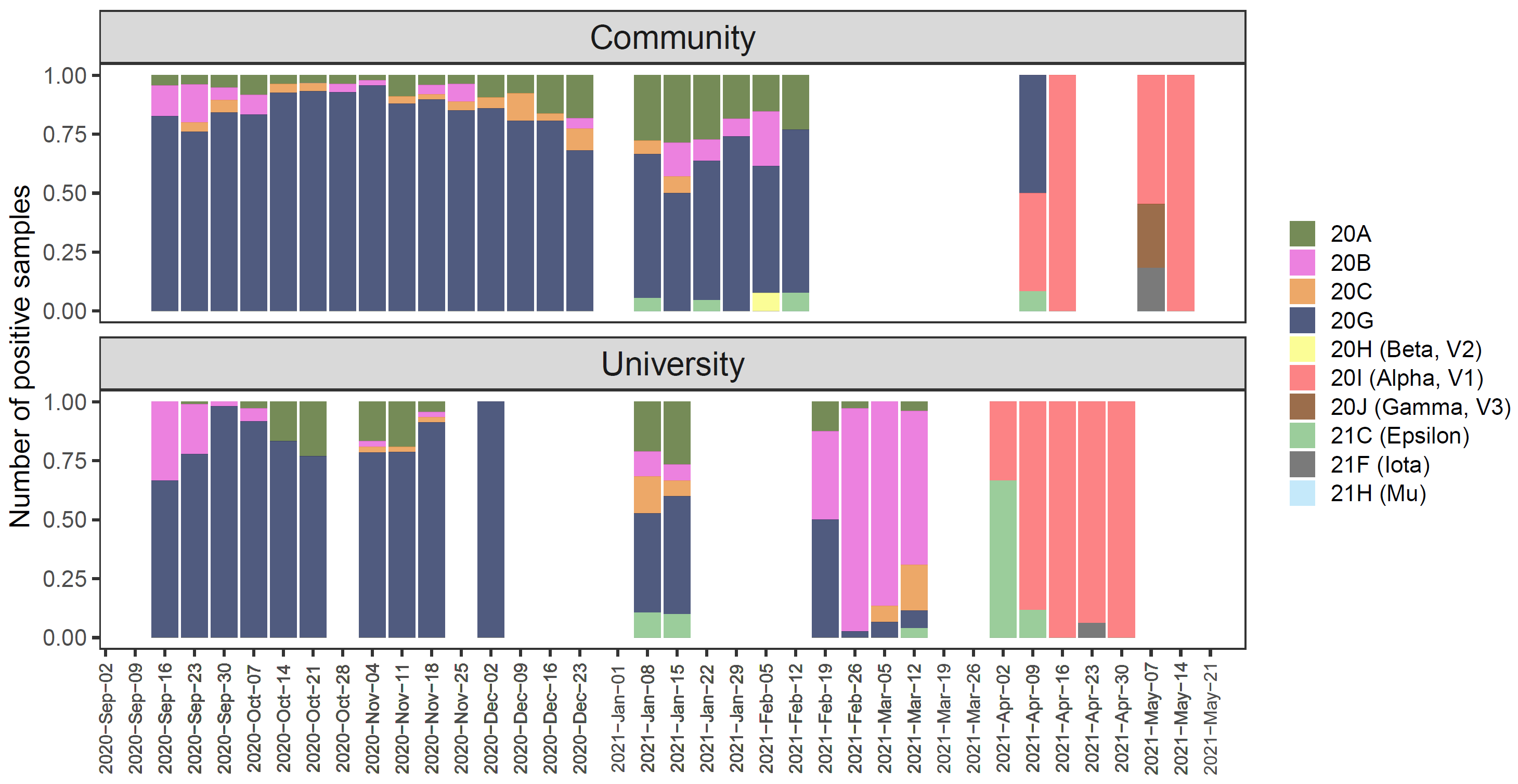


Figure S5. Frequencies of SARS-CoV-2 clades sampled over time for the local community (top) and the University of Idaho (bottom) during the 2020-2021 academic year. Results are shown only for timepoints with a minimum of eight samples.


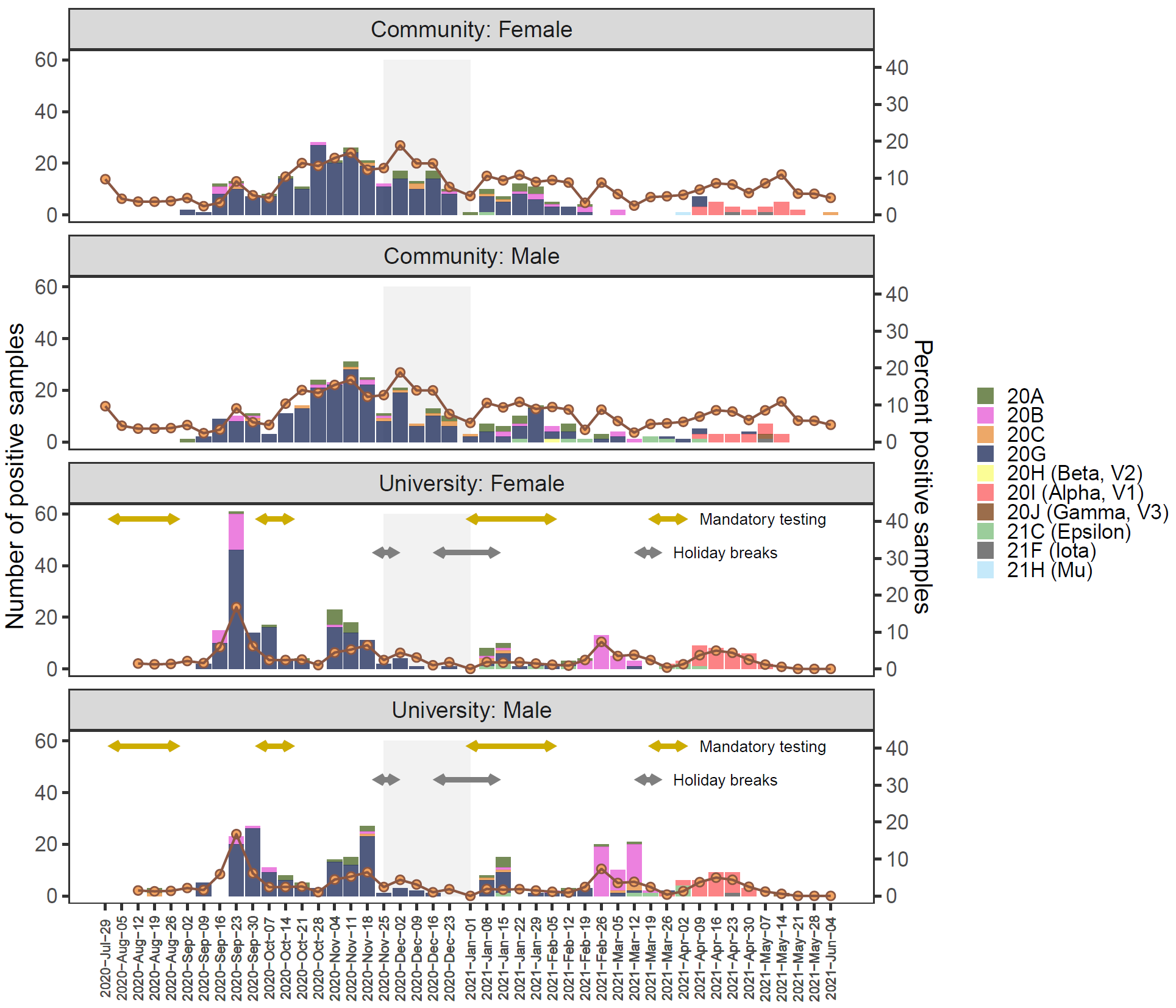


Figure S6. Comparison of counts of SARS-CoV-2 genomes collected from females versus males during the 2020-2021 academic year for the local community (top two plots) or the University of Idaho students, staff, and faculty (bottom two plots). Genomes are colored by Nextstrain clade. Orange line indicates the percent positive samples for both sexes. Light grey shaded region indicates a period of online-only instruction when fewer students were on campus. Double-sided arrows indicate mandatory SARS-CoV-2 testing periods for undergraduates, and University holiday breaks.


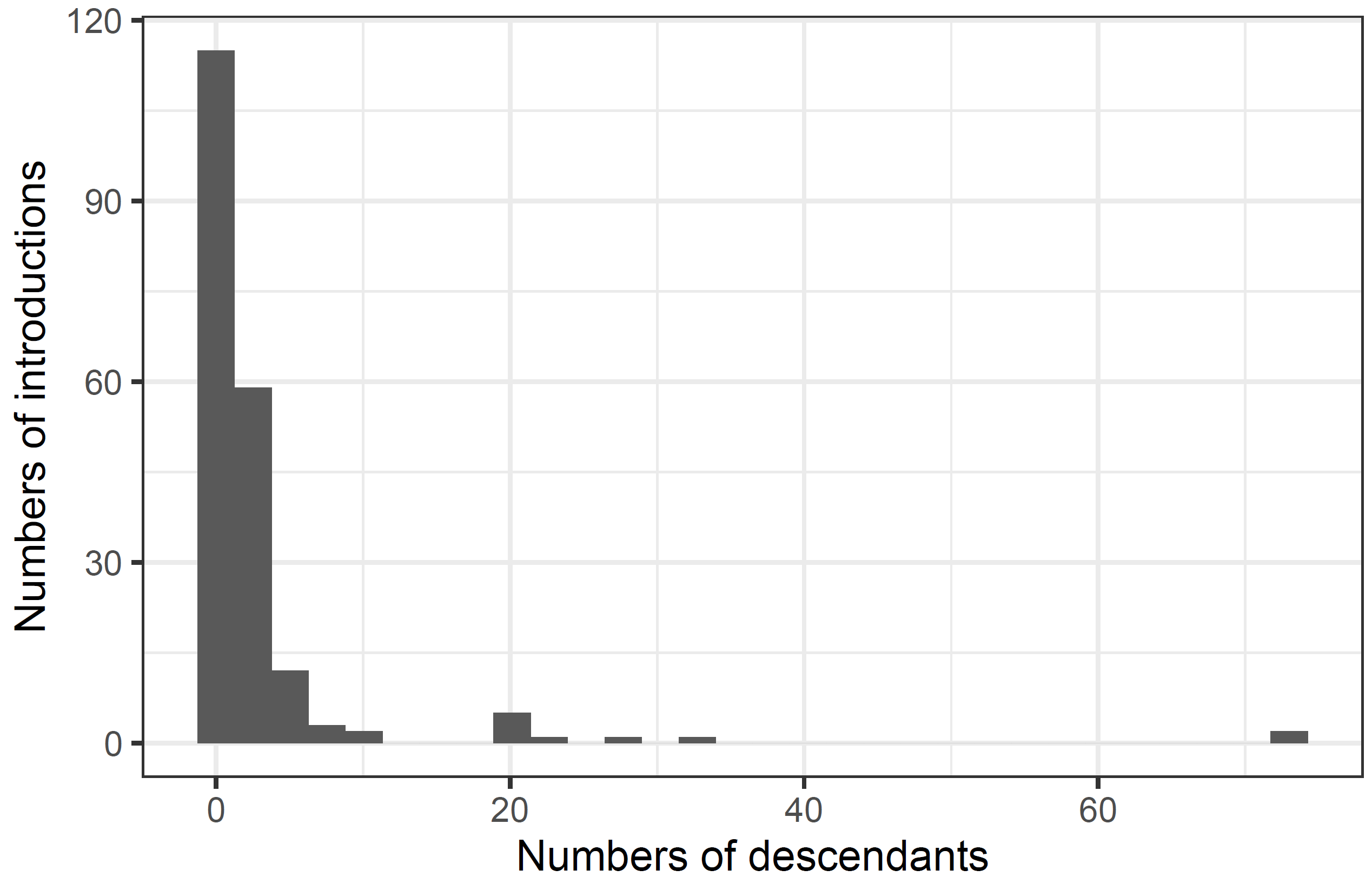


Figure S7. Histogram of the number of descendants for each SARS-CoV-2 introduction into the study populations (excluding one very large introduction that had n=488 descendants) for introductions identified using the UShER phylogeny approach.


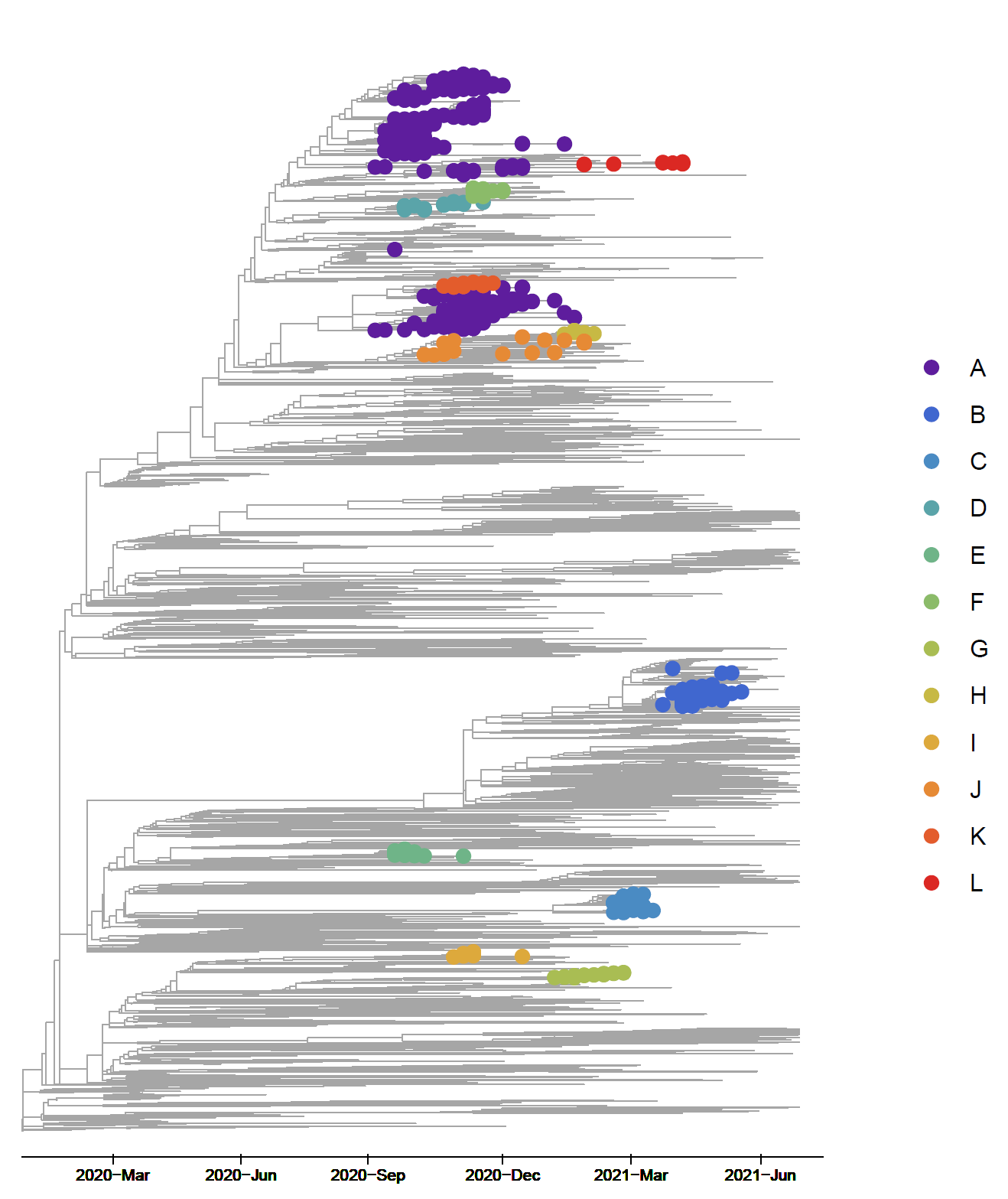


Figure S8. Time-calibrated maximum likelihood phylogeny of SARS-CoV-2 genomes for this study, with color indicating descendants of each of the 12 large post-introduction clades (i.e., introductions which led to >10 sampled descendants) identified using the UShER phylogeny approach. An interactive version of this phylogeny is available at https://nextstrain.org/community/narratives/kimandrews/UofISARSCoV2.


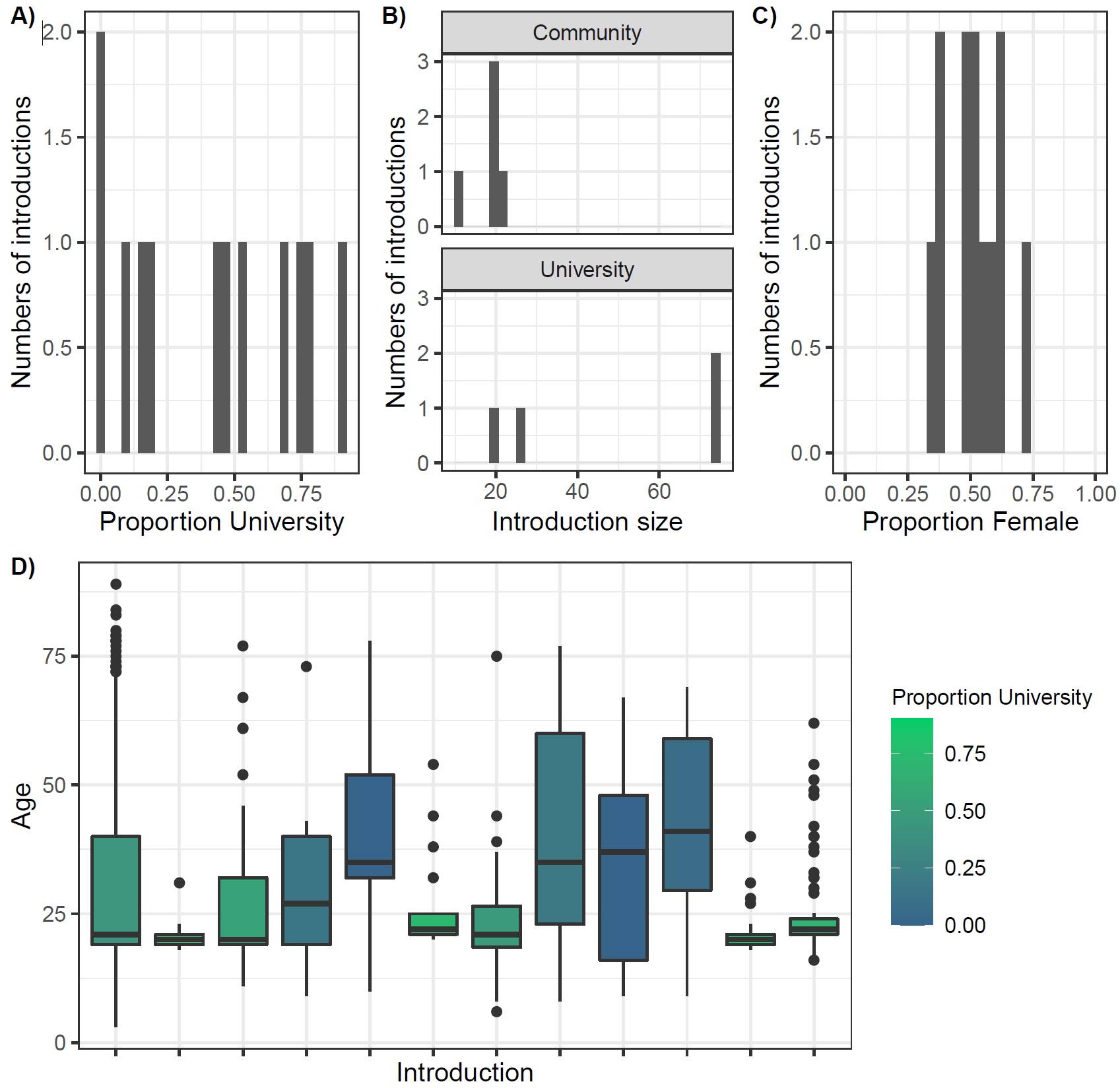


Figure S9. Summary statistics for SARS-CoV-2 introductions that yielded more than 10 descendants, for introductions identified using the UShER phylogeny approach. A) Histogram of the proportion of descendants that were from the University or the local community for each post-introduction clade; B) Histogram of the number of descendants for each introduction, separated by post-introduction clades that were dominated by University or Community samples (>60% of samples from one population), excluding one introduction with a very large number of descendants (n=488 descendants); C) Histogram of the proportion of descendants for each introduction that were from female or male patients; D) Age distribution for each post-introduction clade, plotted in temporal order and colored by the proportion of samples from the University (green) or the local Community (blue).


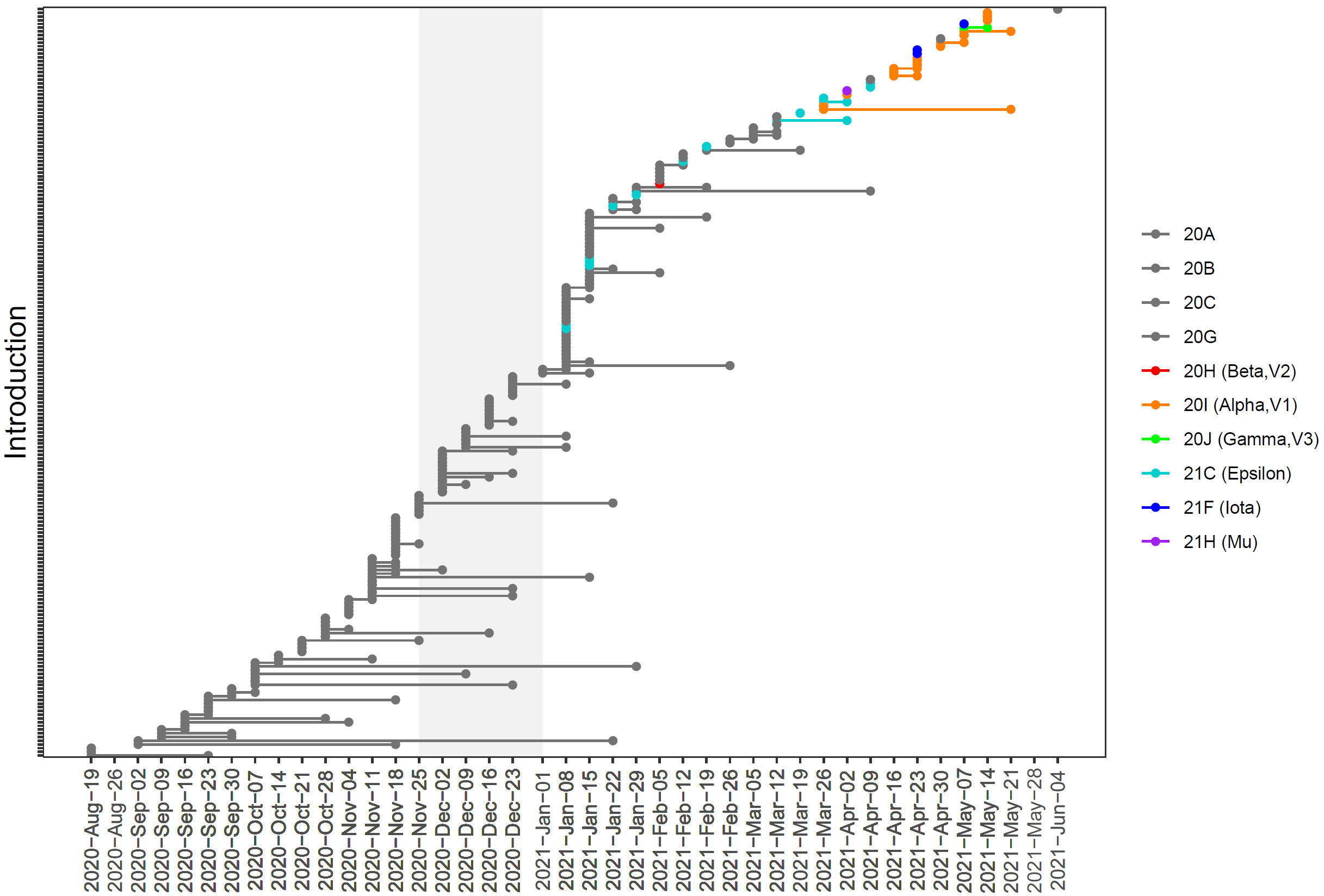


Figure S10. Start and end dates for SARS-CoV-2 introductions reported in Figure 3, with introductions color-coded by variants of concern or variants of interest. Light grey shaded region indicates a period of online-only instruction when fewer students were on campus.

A)


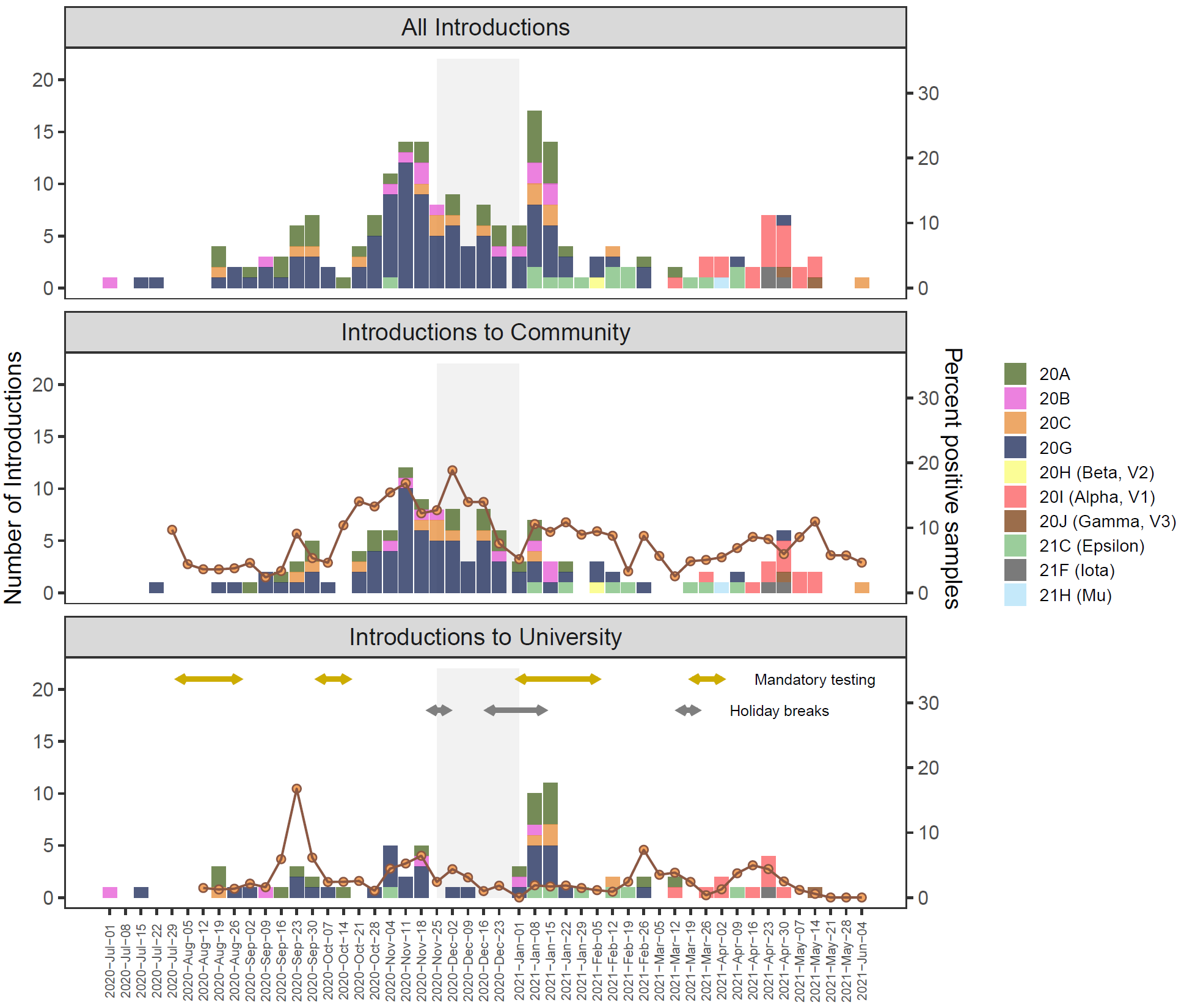


B)
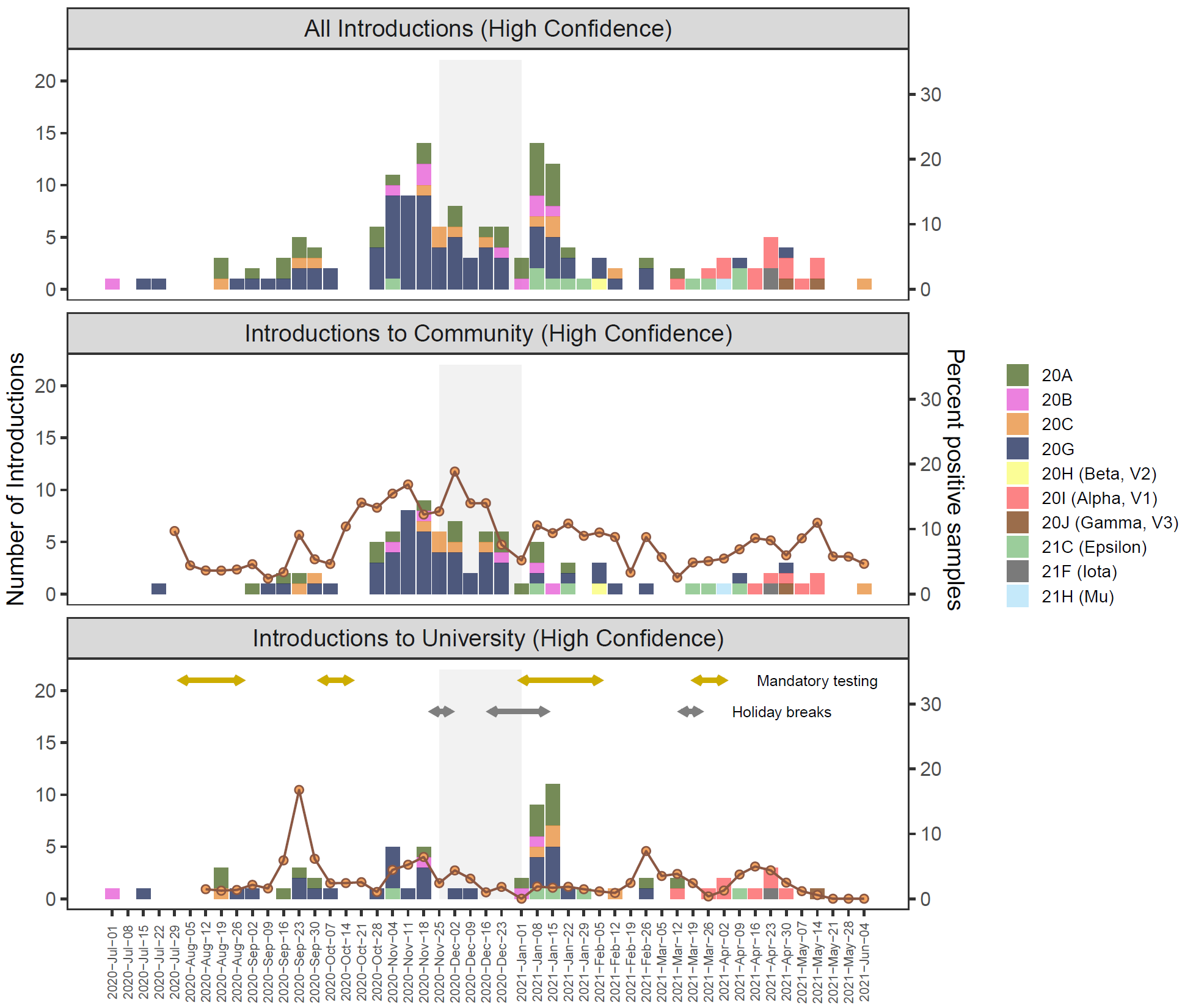


Figure S11. Numbers of introductions of new SARS-CoV-2 variants over time into our focal study populations identified by discrete trait analysis, including A) all introductions identified; B) only high confidence introductions (population assignment confidence > 0.8 for parent and child nodes). For each panel, the top plot = all introductions; middle plot = introductions into local community; bottom plot = introductions into University. Orange lines show SARS-CoV-2 positivity rates are for each recipient population. Light grey shaded region indicates a period of online-only instruction when fewer students were on campus. Double-sided arrows indicate mandatory SARS-CoV-2 testing periods for undergraduates, and University holiday breaks.

A)


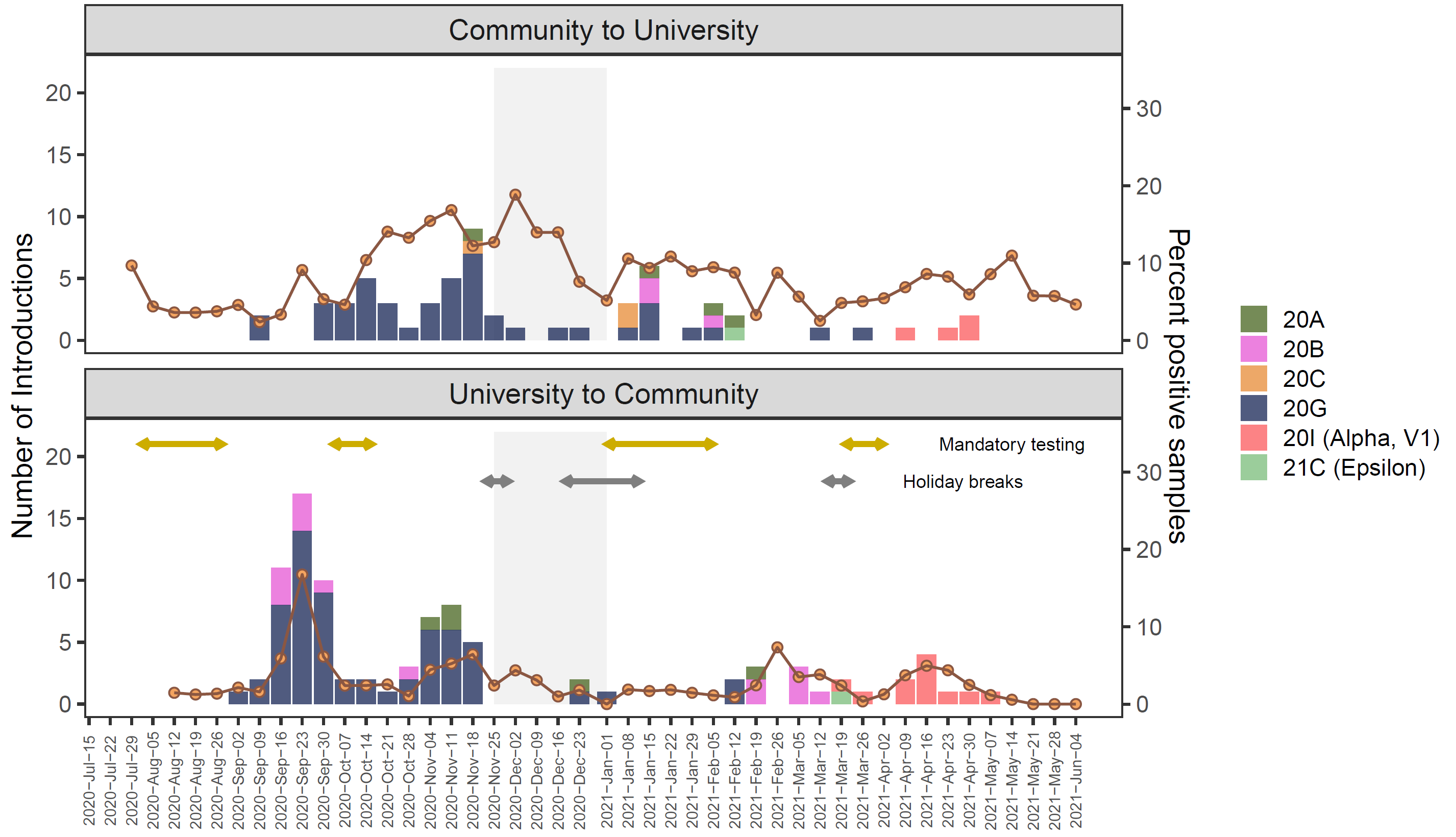


B)


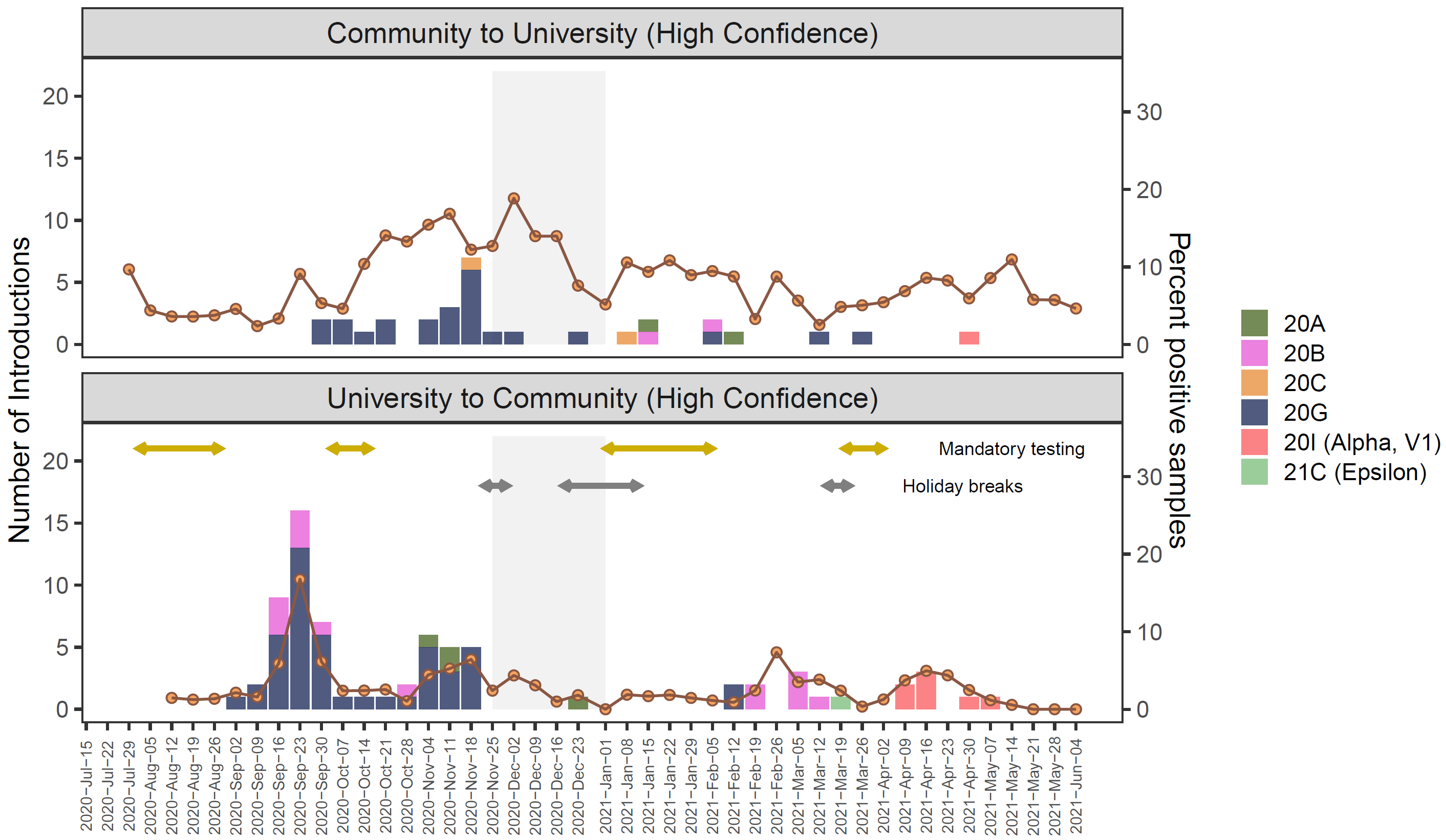


Figure S12. Numbers of SARS-CoV-2 transmissions between the local community and the University of Idaho over time identified by discrete trait analysis, including A) all transmissions identified; B) only high confidence transmissions (population assignment confidence > 0.8 for parent and child nodes). Orange lines show SARS-CoV-2 positivity rates for each source population. Double-sided arrows indicate mandatory SARS-CoV-2 testing periods for undergraduates, and University holiday breaks.


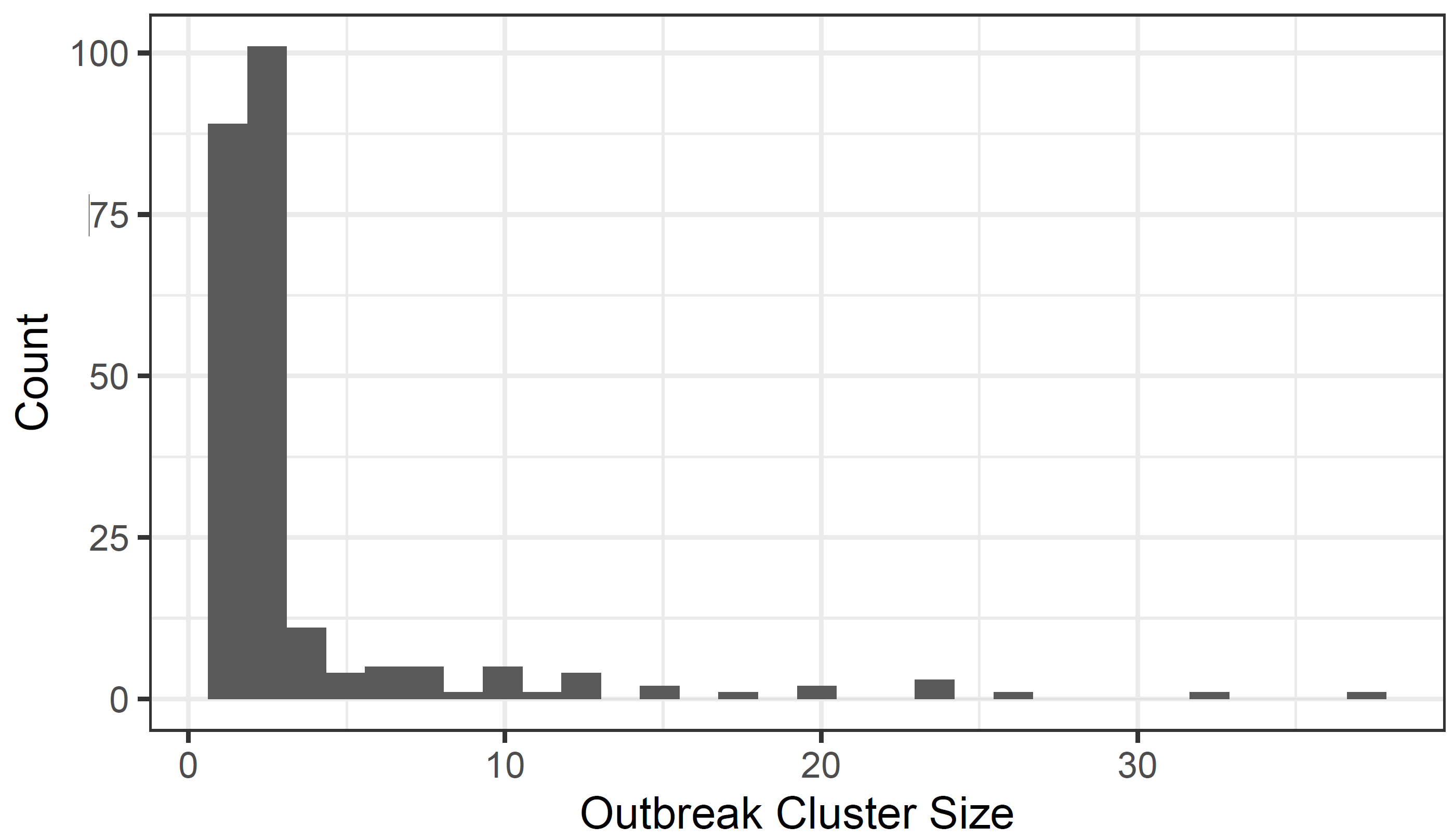


Figure S13. Histogram of SARS-CoV-2 outbreak cluster sizes (i.e., numbers of individuals in each polytomy).


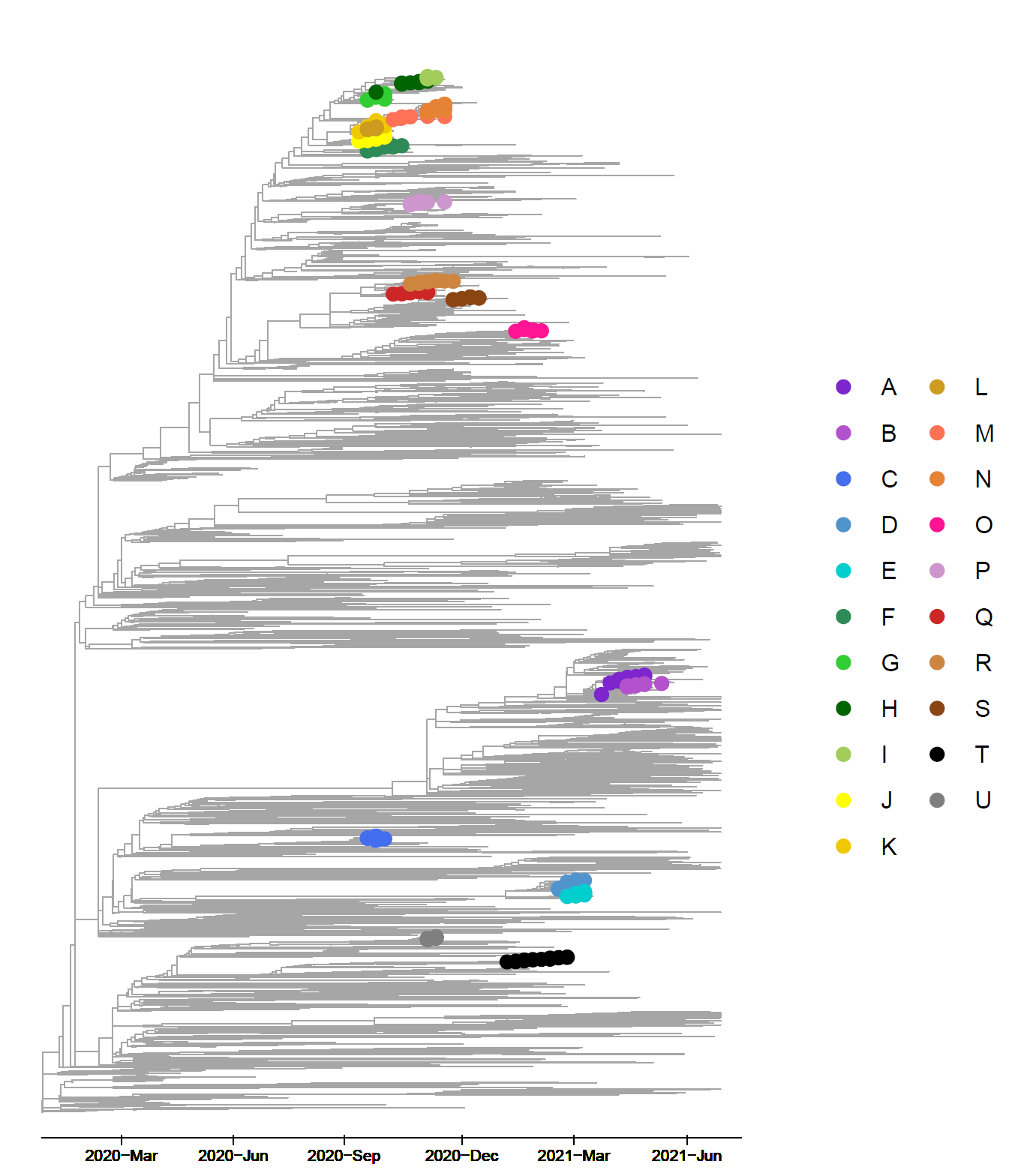


Figure S14. Time-calibrated maximum likelihood phylogeny of SARS-CoV-2 genomes for this study, highlighting the 21 outbreak clusters that had 10 or more individuals. An interactive version of this phylogeny is available at https://nextstrain.org/community/narratives/kimandrews/UofISARSCoV2.


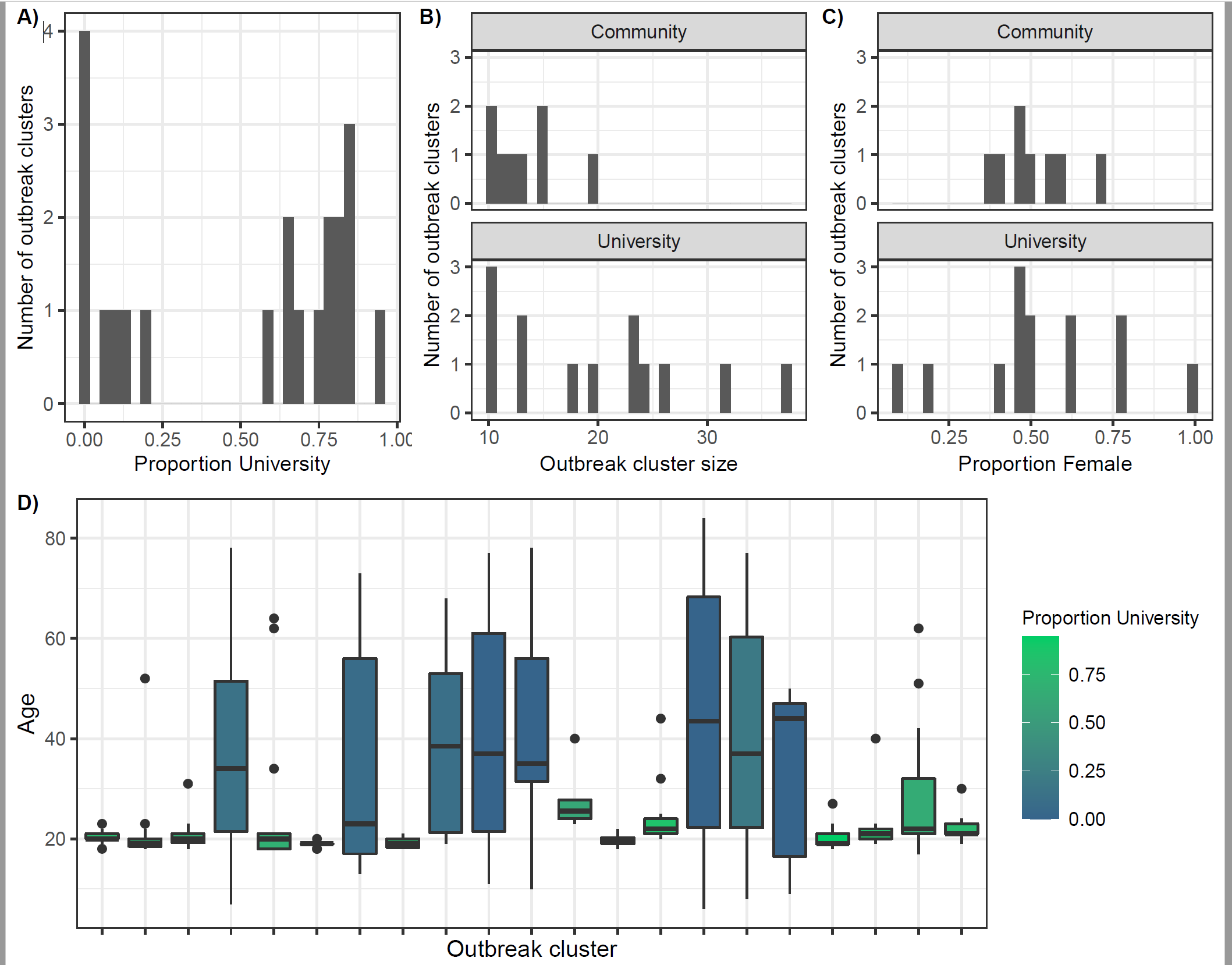


Figure S15. Summary statistics for SARS-CoV-2 outbreak clusters that had 10 or more individuals. A) Histogram of the proportion of individuals that were from the University or the local community for each polytomy; B) Histogram of the number of individuals for each outbreak cluster, plotted separately for clusters that were dominated by University or Community samples (>60% of samples from one population); C) Histogram of the proportion of individuals for each outbreak cluster that were from female or male patients, plotted separately for clusters that were dominated by University or Community samples; D) Age distribution for each outbreak cluster, plotted in temporal order and colored by the proportion of samples from the University (green) or the Community (blue).
